# Dihydroartemisinin alleviates allergic airway inflammation by suppressing the PI3K-AKT signaling axis: integrated network pharmacology and experimental validation

**DOI:** 10.1186/s12931-026-03956-0

**Published:** 2026-10-15

**Authors:** Suidong Ouyang, Daichi Chen, Hangyu Li, Jingyi Deng, Yuqia Xie, Su Chen, Xinlong Lian, Gonghua Huang, Xueqin Huang

**Affiliations:** 1 The First Dongguan Affiliated Hospital, College of Medical Technology, Guangdong Medical University, Dongguan, 523808 China; 2 Dongguan Key Laboratory of Immune Regulation and Prevention of Pulmonary Diseases, Liaobu Hospital of Dongguan City, Dongguan, 523430 China; 3 Guangdong Provincial Key Laboratory of Medical Immunology and Molecular Diagnostics, Guangdong Medical University, Dongguan, 523808 China

**Keywords:** Dihydroartemisinin, Asthma, Network pharmacology, PI3K-AKT pathway

## Abstract

**Background:**

Allergic asthma is a heterogeneous inflammatory airway disorder driven by dysregulated immune responses and epithelial–immune crosstalk. Dihydroartemisinin (DHA), an active derivative of artemisinin, exhibits broad anti-inflammatory and immunomodulatory properties; however, its mechanistic role in allergic asthma remains incompletely defined.

**Methods:**

Network pharmacology was employed to predict potential therapeutic targets and signaling pathways of DHA in asthma. These predictions were validated in a house dust mite (HDM) induced murine asthma model and HDM stimulated human bronchial epithelial (HBE) cells using histopathology, flow cytometry, RT-qPCR, Western blotting, ELISA, and molecular docking.

**Results:**

Network analysis identified nine hub targets and highlighted the PI3K-AKT pathway as a central regulatory axis. In vivo, DHA markedly attenuated airway inflammation, reduced eosinophil and neutrophil infiltration, and decreased Th2 cytokine production. In HDM-stimulated HBE cells, DHA suppressed pro-inflammatory cytokine expression and downregulated EGFR, MMP9, and PTGS2. Mechanistically, DHA inhibited activation of the PI3K-AKT-mTOR signaling cascade both in vitro and in vivo, consistent with molecular docking predictions demonstrating stable binding of DHA to PI3K related targets.

**Conclusions:**

DHA alleviates allergic airway inflammation predominantly through suppression of the PI3K-AKT signaling axis, thereby modulating epithelial inflammatory responses and type 2 immunity. These findings provide mechanistic insight into DHA-mediated immunoregulation and support its potential repositioning as a therapeutic candidate for allergic asthma.

**Supplementary Information:**

The online version contains supplementary material available at https://doi.org/10.1186/s12931-026-03956-0.

## Introduction

Allergic asthma is a chronic and heterogeneous inflammatory airway disease characterized by persistent airway inflammation, reversible airflow limitation, mucus hypersecretion, and progressive airway remodeling. The pathogenesis of asthma involves complex interactions between immune cells including mast cells, eosinophils, and T lymphocytes and structural airway cells such as epithelial cells, which together orchestrate dysregulated immune responses and epithelial-immune crosstalk [1]. Upon allergen exposure, the excessive release of inflammatory mediators, including leukotrienes, prostaglandins, histamine, and cytokines, promotes airway smooth muscle contraction, vascular permeability, and goblet cell hyperplasia, ultimately leading to airway narrowing and respiratory dysfunction [2]. In addition, sustained overproduction of pro-inflammatory cytokines such as TNF-α, IL-1β, and IL-6, together with increased reactive oxygen species (ROS), exacerbates oxidative stress and epithelial injury, thereby amplifying inflammatory signaling and perpetuating chronic airway inflammation [3, 4].

Despite significant advances in asthma management, the global prevalence of the disease continues to rise, posing a considerable public health burden [5]. Current therapeutic strategies mainly rely on inhaled corticosteroids (ICS), bronchodilators, and biologic agents targeting type 2 inflammatory pathways [6–8]. Although these treatments effectively alleviate symptoms in many patients, they rarely achieve long-term disease modification and may cause adverse effects with prolonged use, including metabolic disturbances, osteoporosis, hepatotoxicity, and gastrointestinal complications [9]. Moreover, corticosteroid resistance and heterogeneous therapeutic responses remain major clinical challenges, underscoring the need for novel therapeutic strategies that target upstream molecular mechanisms driving airway inflammation and remodeling [6, 10].

In recent years, bioactive compounds derived from natural products and traditional herbal medicines have attracted increasing attention due to their multi-target pharmacological properties and relatively favorable safety profiles [11–13]. Dihydroartemisinin (DHA), the principal active metabolite of artemisinin isolated from *Artemisia annua*, has been widely used as a first-line antimalarial agent [14, 15]. Mechanistically, DHA exerts antiparasitic activity by disrupting parasite membrane integrity, impairing mitochondrial function, and inducing ROS mediated oxidative stress [16]. Beyond its antimalarial effects, accumulating evidence suggests that DHA possesses broad anti-inflammatory and immunomodulatory activities. Previous studies have demonstrated that DHA suppresses tumor cell proliferation through inhibition of mTOR signaling [17, 18], alleviates experimental colitis by downregulating NF-κB activation [19, 20], and mitigates psoriasis-like inflammation by reducing the production of pro-inflammatory cytokines such as IL-17 A, IL-23, and TNF-α [10, 21]. Additionally, DHA has been reported to restore immune homeostasis by modulating T-cell subsets, including the regulation of Treg/Th17 balance [22]. These findings suggest that DHA may target conserved inflammatory signaling pathways involved in chronic inflammatory diseases, including asthma.

Among the signaling pathways implicated in asthma pathogenesis, the phosphatidylinositol-3-kinase/protein kinase B (PI3K-AKT) signaling pathway has emerged as a central regulator of airway inflammation, epithelial barrier function, and immune cell activation [23, 24]. Aberrant activation of PI3K-AKT signaling contributes to Th2-dominant immune responses, epithelial-derived alarmin release, mucus hypersecretion, and corticosteroid insensitivity, thereby playing a critical role in asthma development and progression [25, 26]. Consequently, targeting the PI3K-AKT pathway has been proposed as a promising therapeutic strategy for allergic asthma. However, whether DHA exerts protective effects against allergic airway inflammation through modulation of PI3K-AKT centered signaling networks remains largely unclear.

Given the complex pathogenesis of asthma and the pleiotropic pharmacological properties of DHA, traditional single-target approaches are insufficient to comprehensively elucidate its mechanisms of action. Network pharmacology, an emerging systems-level strategy integrating pharmacology, bioinformatics, and network biology, provides an effective framework for investigating multi-target drug–disease interactions [27, 28]. By constructing integrative drug–target–pathway networks, this approach enables the identification of key regulatory nodes and signaling pathways underlying the therapeutic effects of bioactive compounds [29]. Nevertheless, computational predictions require experimental validation to ensure their biological relevance [30, 31].

In the present study, we integrated network pharmacology analysis with in vivo and in vitro experimental validation to systematically investigate the molecular mechanisms by which DHA attenuates allergic airway inflammation. Our findings identify the PI3K-AKT signaling pathway as a critical regulatory axis mediating the anti-inflammatory effects of DHA, providing mechanistic insight into its therapeutic potential and supporting its development as a promising candidate for the treatment of allergic asthma.

## Materials and methods

### Prediction of DHA targets

The canonical SMILES structure of dihydroartemisinin (DHA) was retrieved from PubChem (https://pubchem.ncbi.nlm.nih.gov/). The SMILES string was uploaded to the SwissTargetPrediction database (http://www.swisstargetprediction.ch/) to predict potential pharmacological targets.

### Identification of asthma associated targets

Asthma related targets were systematically collected from GeneCards (https://www.genecards.org/), OMIM (https://www.omim.org/), and the Therapeutic Target Database (TTD, http://db.idrblab.net/ttd/). After removal of duplicates, only human-specific targets with strong relevance scores were included. The intersection between DHA-predicted and asthma related targets was defined as putative therapeutic targets of DHA against asthma.

### Protein-protein interaction network and functional enrichment

The overlapping targets were uploaded to STRING (https://string-db.org/) to construct a protein–protein interaction (PPI) network, with species restricted to *Homo sapiens*, medium confidence score (0.4), and FDR correction at 5%. Network visualization and hub gene identification (based on degree centrality and other topological features) were performed using Cytoscape (v3.9.0). Gene Ontology (GO) enrichment (biological processes, cellular components, molecular functions) and Kyoto Encyclopedia of Genes and Genomes (KEGG) pathway analyses were conducted via the Weishengxin platform (http://www.bioinformatics.com.cn/), with statistical significance set at adjusted *p* < 0.05.

### Molecular docking and molecular dynamics simulation

Crystal structures of candidate target proteins were obtained from the Protein Data Bank (PDB, https://www.rcsb.org/). The 2D structure of DHA was retrieved from PubChem, converted to 3D using ChemDraw/Chem3D, and energy-minimized. Molecular docking was conducted using AutoDock Vina, and binding interactions were visualized with PyMOL and Discovery Studio. Complexes with high binding affinity underwent molecular dynamics simulations using GROMACS to evaluate stability and interaction dynamics under physiological conditions.

### Animals and housing

Twenty male SPF C57BL/6 mice (8 weeks old, 20 ± 2 g) were purchased from the Guangdong Experimental Animal Center (Guangzhou, China). Mice were maintained at the Guangdong Medical University Animal Facility under controlled temperature (23 ± 2 °C), relative humidity (65% ± 5%), and a 12-h light/dark cycle. All procedures were approved by the Institutional Animal Care and Use Committee (IACUC) of Guangdong Medical University and complied with the NIH Guide for the Care and Use of Laboratory Animals (Approval No.: GDY2002326).

### HDM induced asthma model and DHA administration

Mice were randomly assigned into four groups (*n* = 5 per group): (i) Control (PBS), (ii) HDM model, (iii) HDM + DHA (30 mg/kg), and (iv) HDM + DHA (100 mg/kg). On days 0 and 2, mice received intranasal instillation of house dust mite (HDM; 200 µg in PBS; Greer Laboratories, USA). From days 14–18, HDM challenges (50 µg/day) were repeated intranasally. DHA (Aladdin, Shanghai, China) was administered intranasally at 30 or 100 mg/kg on days 2 and 14–20. On day 21, mice were anesthetized, and bronchoalveolar lavage fluid (BALF), lung tissue, and serum were collected. Three independent experiments were performed, with five mice per group in each experiment.

### BALF collection and flow cytometry of lung leukocytes

BALF was collected by instilling 1 mL ice-cold PBS via the trachea, followed by gentle aspiration. Samples were centrifuged at 300 × g for 10 min at 4 °C, and cell pellets were resuspended for analysis. For lung leukocytes, tissues were excised, minced, and digested with collagenase IV (1 mg/mL; Life Technologies) in RPMI-1640 supplemented with 5% FBS for 45 min at 37 °C. Suspensions were filtered through 70 μm strainers, followed by Percoll (38%) density gradient centrifugation. Red blood cells were lysed with ACK buffer, and leukocytes were subjected to flow cytometric analysis.

Cells were stained with fluorochrome-conjugated monoclonal antibodies in PBS with 1% FBS for 30 min on ice. For intracellular cytokine staining, leukocytes were stimulated with PMA (50 ng/mL) and ionomycin (1 µM) in the presence of GolgiStop (BD Biosciences) for 5 h. Following fixation and permeabilization, intracellular staining was performed per the manufacturer’s instructions. Data were acquired on an LSRFortessa™ X-20 cytometer (BD Biosciences) and analyzed with FlowJo (v10.7).

### Histopathological analysis

After PBS perfusion, lung tissues were fixed in 4% paraformaldehyde overnight, paraffin-embedded, and sectioned (5 μm). Sections were stained with hematoxylin and eosin (H&E) for general histopathology and periodic acid-Schiff (PAS) for goblet cell hyperplasia and mucus secretion. Slides were examined under light microscopy.

### Cell culture and treatment

Human bronchial epithelial cells (16HBE; ATCC, USA) were cultured in DMEM (Gibco) with 10% FBS, 100 U/mL penicillin, and 100 µg/mL streptomycin at 37 °C in 5% CO₂. For experiments, cells (1 × 10⁶/well) were seeded in 6-well plates and treated with DHA (20 µM) or vehicle (0.1% DMSO) for 24 h (protein extraction) or 6 h (RNA extraction).

### ELISA

Serum was collected from the right ventricle and centrifuged (5000 rpm, 10 min, 4 °C). Cytokines (IL-4, IL-5, IL-13) and total IgE were quantified in serum and BALF using commercial ELISA kits (Invitrogen, USA). For in vitro assays, 16HBE cells were pretreated with DHA (20 µM, 2 h), followed by HDM stimulation (100 µg/mL, 24 h). Supernatants were centrifuged and analyzed by ELISA. All samples were assayed in duplicate.

### RT-qPCR

Total RNA was extracted from lung tissues and 16HBE cells using Trizol (Invitrogen). 1 µg of RNA was reverse-transcribed with the PrimeScript™ RT Reagent Kit (TaKaRa, Japan). RT-qPCR was performed with SYBR Green Master Mix (Yeasen, China) on a LightCycler^®^ 96 (Roche, Germany). GAPDH served as the reference gene, and relative expression was calculated using the 2^^–ΔΔCt^ method. Primer sequences are listed in Table 1.

**Table 1:** List of primers used for RT-qPCR

Gene	Species	Forward primer	Reverse primer
*Tnfα*	Mouse	CAGGCGGTGCCTATGTCTC	CGATCACCCCGAAGTTCAGTAG
*Il6*	Mouse	ATGAAGTTCCTCTCTGCAAGAGACT	CACTAGGTTTGCCGAGTAGATCTC
*Gapdh*	Mouse	AGGTCGGTGTGAACGGATTTG	TGTAGACCATGTAGTTGAGGTCA
*Il1β*	Mouse	CCCTCACACTCAGATCATCTTCT	GCTACGACGTGGGCTACAG
*Ifnγ*	Mouse	CGGAGAGGAGACTTCACAGAG	CATTTCCACGATTTCCCAGA
*Mmp9*	Mouse	CTGGACAGCCAGACACTAAAG	CTCGCGGCAAGTCTTCAGAG
*Egfr*	Mouse	GCGTTCGGCACGGTGTATAA	GGCTTTCGGAGATGTTGCTTC
*Il5*	Mouse	GCAATGAGACGATGAGGCTTC	CCTCATCGAGCCCACAGACAT
*Il13*	Mouse	GCAATGGCATCTTGGTGATCCT	GCAACGGCAGCATCTTGGAGT
*Muc5b*	Mouse	AGTGCCACAGCAACAGTGAC	CAGCTCCTCTGGGTTCACCT
*Muc5ac*	Mouse	TCTGGCCTGTGTGGACTTGAT	CCTCGTCCTCGAAGTAGCGT
*IL17A*	Human	ACAGTGAAGGCAGCGGTACT	GCTCAGAGTCCAGGGTGAAG
*IL1β*	Human	GTACGATCACTGAACTGCACGC	CACGCAGGACAGGTACAGATTC
*IL4*	Human	TCACCAGCACCTTTGTCCAC	CGCTTGTGTTCTTTGGAGCA
*IL5*	Human	GCTTCCACAGGCAATGAGAC	AGGCTTTCCAGGCAGACAGG
*IL6*	Human	GGAACAAGCCAGAGCTGTGC	TGCCGAAGAGCCCTCAGG
*TNFα*	Human	CTTCTCCTTCCTGATCGTGC	GCTGGTTATCTCTCAGCTCCA
*IFNγ*	Human	GGGTTCTCTTGGCTGTTACTG	TTCTGTCACTCTCCTCTTTCCA
*GAPDH*	Human	CAACAGCGACACCCACTCCT	CACCCTGTTGCTGTAGCCAAA

### Western blotting

Proteins from lung tissues and 16HBE cells were extracted with RIPA buffer (Beyotime, China) containing protease and phosphatase inhibitors (Roche). Protein concentrations were determined using the BCA assay (Thermo Fisher, USA). Equal amounts of protein (30 µg for tissues, 10 µg for cells) were resolved by SDS-PAGE and transferred to PVDF membranes. After blocking with 5% non-fat milk, membranes were incubated overnight at 4 °C with primary antibodies: PI3K (rabbit Ab, Cat No. 4257), P-S6 (rabbit Ab, Cat No. 4858), AKT (rabbit Ab, Cat No. 4685), β-actin (rabbit Ab, Cat No. 4970 S), TNF-a (rabbit Ab, Cat No. 11948), IL-1β (mouse Ab, Cat No. 12242), P65(rabbit Ab, Cat No. 8242)from Cell Signaling Technology Inc. USA; NRF2 (mouse Ab, Cat No. 266504-1-Ig), MMP9 (rabbit Ab, Cat No. 27306-1-AP), EGFR (rabbit Ab, Cat No. 30847-1-AP), PTGS2 (mouse Ab, Cat No. 66351-1-Ig) from Proteintech. USA. After incubation with HRP-conjugated secondary antibodies, bands were visualized using ECL reagents (Millipore, USA) and quantified by ImageJ software.

### Statistical analysis

Data were analyzed using GraphPad Prism 8. Results are expressed as mean ± standard deviation (SD). Comparisons between two groups were performed using unpaired Student’s *t*-tests, and multiple group comparisons were analyzed by one-way ANOVA followed by Tukey’s post hoc test. A *p* < 0.05 was considered statistically significant.

## Results

### Network pharmacology analysis identifies core DHA targets associated with asthma

**Fig. 1:**
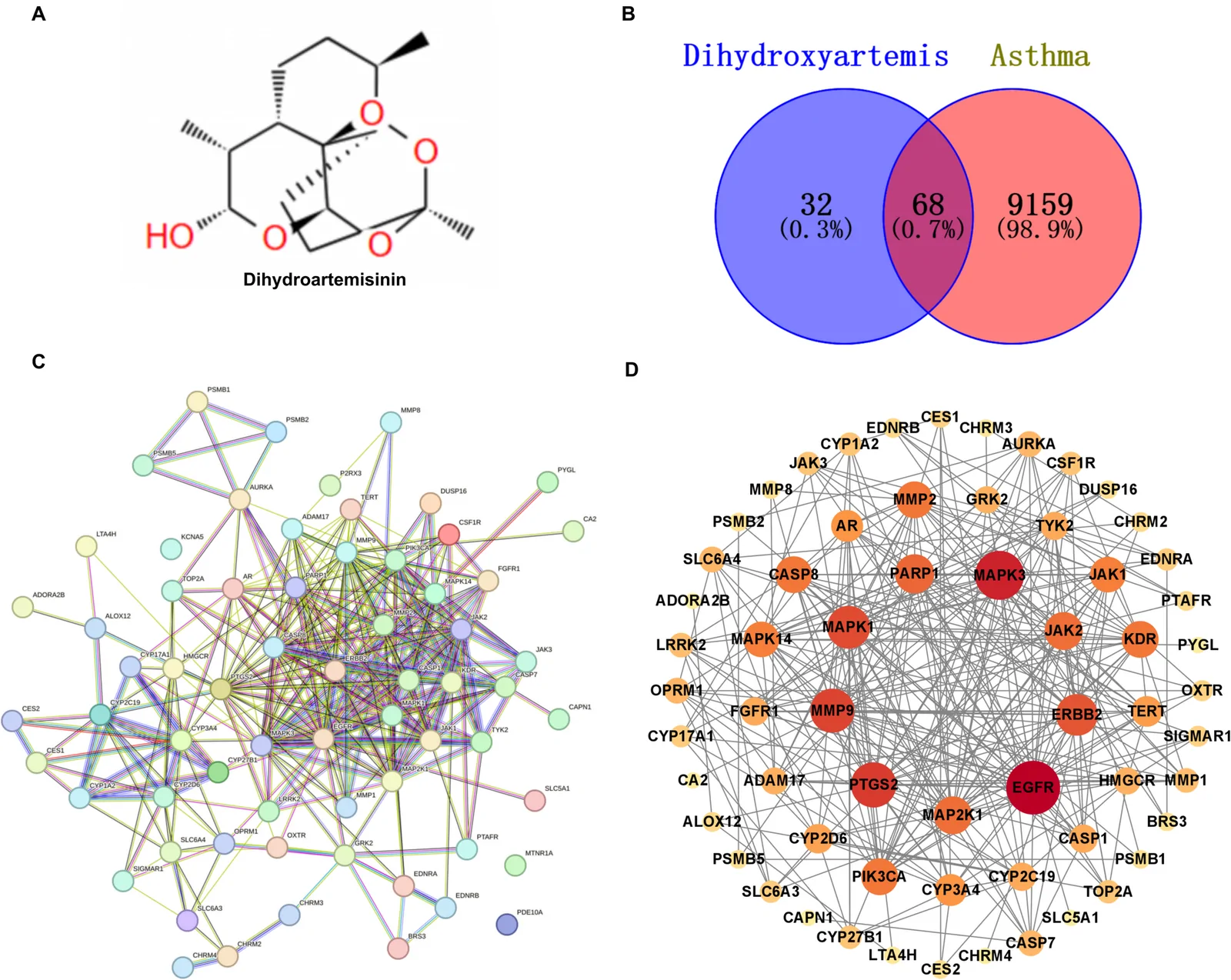
Identification of potential targets for DHA in asthma treatment. **A**. Chemical structure diagram of DHA; **B**.Venn diagram of overlapping targets between DHA and asthma; **C**. PPI analysis of overlapping targets between DHA and asthma; **D**. Network diagram of overlapping targets between DHA and asthma

The molecular structure of DHA was obtained from the PubChem database (Fig. 1A). To systematically elucidate the potential molecular mechanisms underlying the therapeutic effects of DHA in asthma, its candidate targets were predicted using the SwissTargetPrediction platform, yielding 100 putative targets based on structural similarity and reported bioactivity. In parallel, asthma-related genes were comprehensively collected from the GeneCards, Therapeutic Target Database (TTD), and PharmGKB databases. After data integration and removal of duplicate entries, 9,227 asthma-associated targets were retained for subsequent analysis. Intersection analysis between DHA-predicted targets and asthma-related targets was performed using the Venny 2.1 online tool, and the overlapping targets were visualized by a Venn diagram (Fig. 1B), representing potential therapeutic targets of DHA in asthma. These intersecting targets were then imported into the STRING database to construct a protein-protein interaction (PPI) network, allowing systematic evaluation of their functional connectivity and interaction patterns (Fig. 1C). The PPI network was further analyzed using Cytoscape software to identify key regulatory nodes. Topological analysis based on degree centrality revealed nine hub genes: EGFR, MAPK3, PTGS2, MMP9, MAPK1, ERBB2, JAK2, PARP1, and MAP2K1, that exhibited high connectivity within the network (Fig. 1D; Table 2). Notably, these core targets are predominantly implicated in inflammatory signaling cascades, immune response regulation, and airway remodeling processes. Collectively, these findings suggest that DHA may exert its anti-asthmatic effects through coordinated modulation of multiple interconnected signaling pathways rather than through a single molecular target.

**Table 2:** Topological analysis of the DHA-asthma target network

Name	Betweenness Centrality	Closeness Centrality	Degree
EGFR	0.18879197404424597	0.648936170212766	32
MAPK3	0.09752493953906524	0.5865384615384616	28
PTGS2	0.13717323836118545	0.5754716981132075	25
MMP9	0.06438431484221706	0.5350877192982456	24
MAPK1	0.04627912366416698	0.5495495495495495	23
ERBB2	0.033887039514060434	0.5495495495495495	22
PARP1	0.02227880283119333	0.5258620689655172	20
JAK2	0.03310524260956936	0.5398230088495575	19
MAP2K1	0.014714330411474671	0.49593495934959353	19

Note: The higher the values of BC, CC and degree, the more important the node

### GO and KEGG enrichment analyses reveal PI3K-AKT entered signaling in DHA mediated asthma regulation

**Fig. 2:**
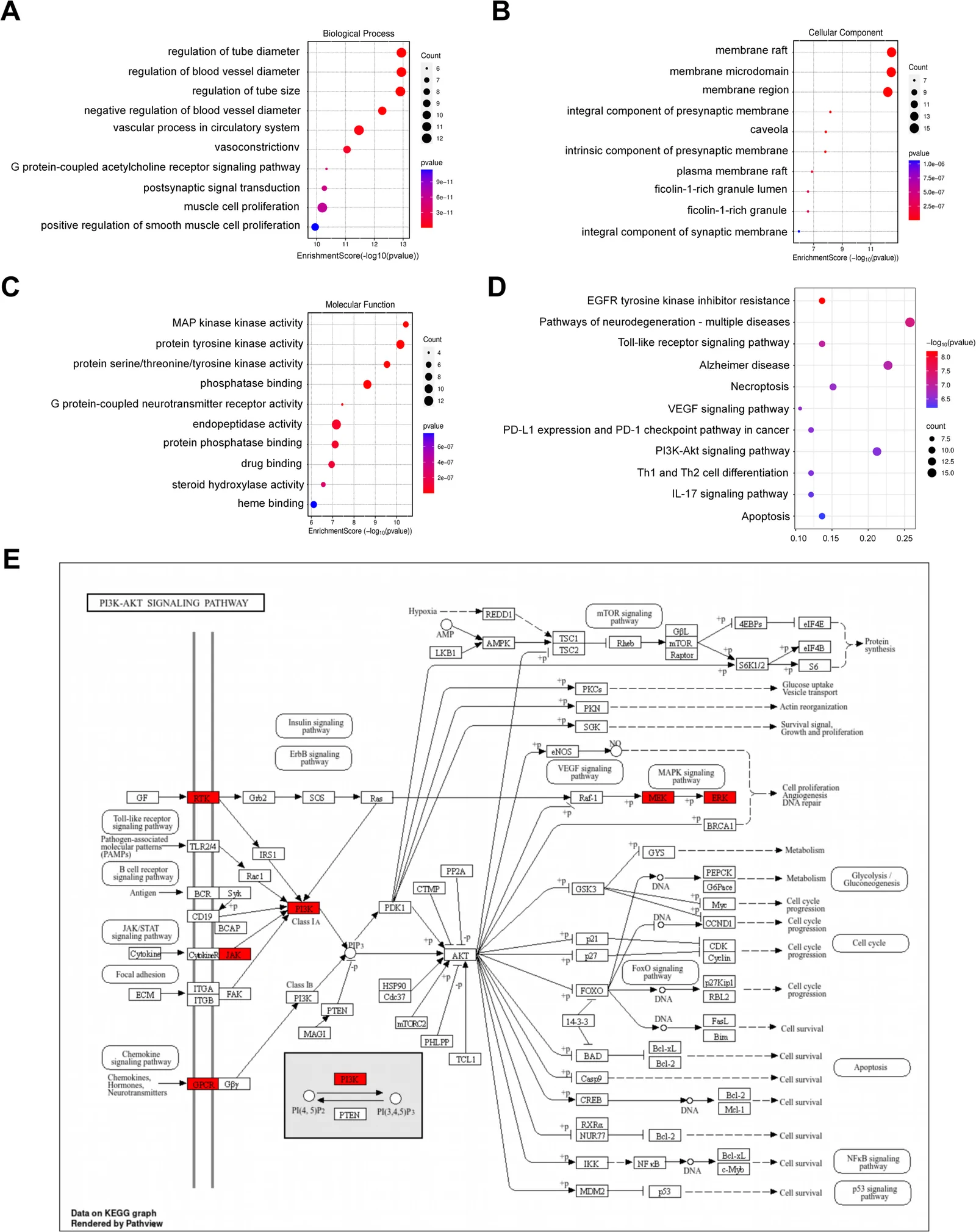
Analysis and validation of biological functions between DHA and asthma. **A-C**. GO functional enrichment analysis of DHA treatment in asthma; **D**. KEGG pathway analysis of DHA treatment in asthma. (**E**) PI3K-AKT signaling pathway indicator diagram

GO enrichment analysis was performed to characterize the functional attributes of the intersecting DHA-asthma targets across biological processes (BP), cellular components (CC), and molecular functions (MF). In the BP category, these targets were predominantly enriched in processes related to regulation of vascular tone and lumen diameter, G protein-coupled acetylcholine receptor signaling, and muscle cell proliferation, all of which are closely associated with airway hyperresponsiveness and structural remodeling in asthma (Fig. 2A). Cellular component analysis revealed significant enrichment in integral components of the membrane, presynaptic membranes, and plasma membrane rafts, indicating that the majority of these targets are membrane associated proteins involved in signal transduction (Fig. 2B). Consistently, MF enrichment analysis demonstrated overrepresentation of kinase-related activities, including MAP kinase activity, protein tyrosine kinase activity, and G protein-coupled receptor activity, highlighting the central role of phosphorylation-dependent signaling cascades in DHA-mediated regulation (Fig. 2C).

To further explore the signaling pathways involved, KEGG pathway enrichment analysis was conducted. The intersecting targets were significantly enriched in multiple inflammation and signal transduction related pathways, including the PI3K-AKT signaling pathway, EGFR tyrosine kinase inhibitor resistance, and the Toll-like receptor signaling pathway (Fig. 2D). These enriched pathways showed a strong convergence on the PI3K-AKT signaling axis, indicating that PI3K-AKT may function as a central regulatory hub through which DHA modulates airway inflammation and immune responses in asthma (Fig. 2E).

### Molecular docking and dynamics simulations validate stable interactions between DHA and key asthma related targets

**Fig. 3:**
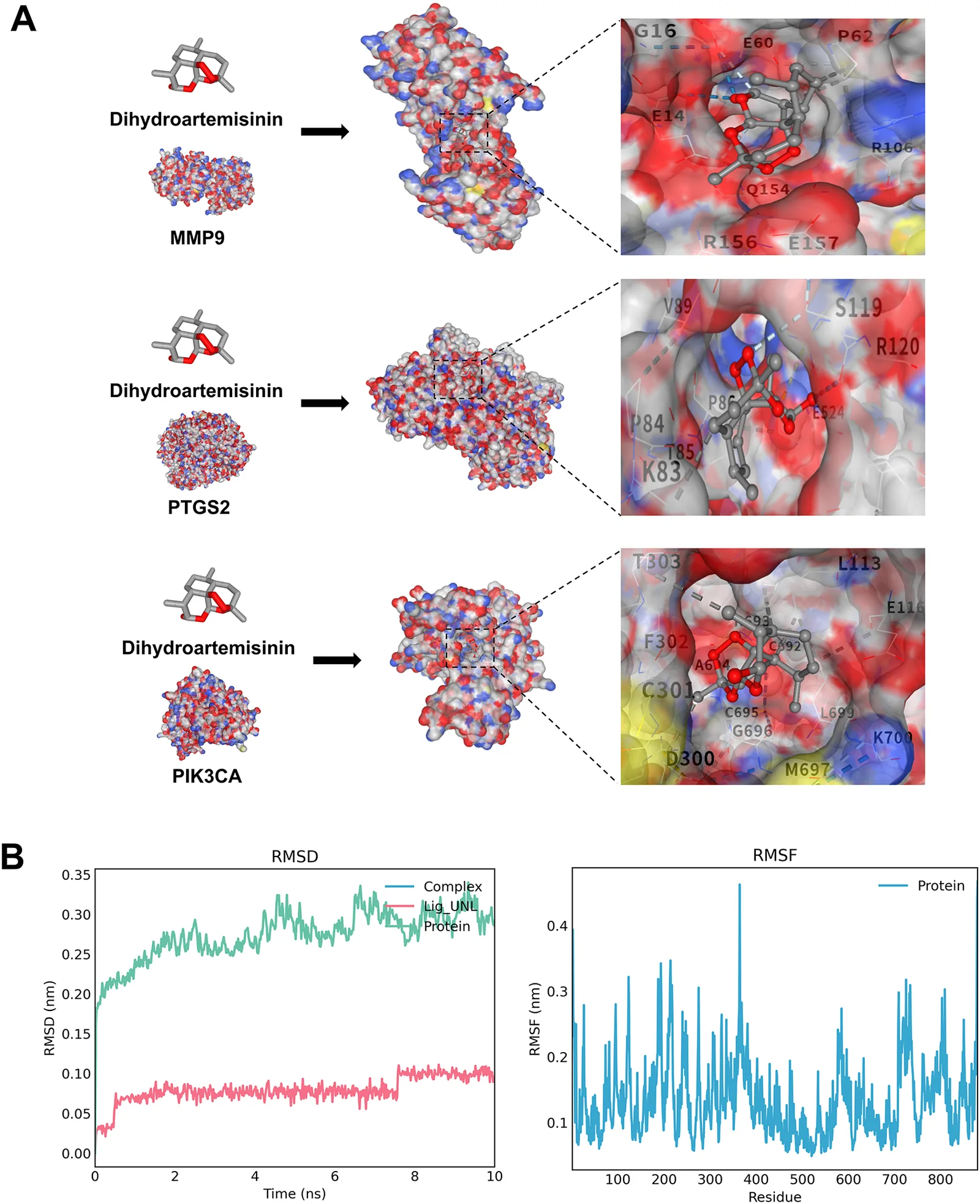
Molecular docking and molecular dynamics analysis. (**A**) Molecular docking analysis between DHA and MMP9/PTGS2/PI3KCA; (**B**) Molecular dynamics analysis between DHA and PI3KCA

Molecular docking analysis was conducted to evaluate the binding potential of DHA with selected core targets identified from network pharmacology analysis. The results revealed favorable binding affinities between DHA and MMP9, PTGS2, and PIK3CA, as evidenced by low binding energies and well-defined docking poses occupying the active or functional pockets of the target proteins (Fig. 3A).

To further investigate the stability and dynamic behavior of these ligand-protein interactions, MD simulations were conducted, with particular focus on the DHA-PIK3CA complex (Fig. 3B). The RMSD analysis indicated that the system gradually stabilized during the simulation, reaching equilibrium after approximately 4 ns. Both the protein backbone and ligand conformations maintained stable trajectories thereafter, suggesting a structurally equilibrated and stable complex. RMSF analysis further showed that most amino acid residues exhibited limited fluctuations, reflecting overall structural rigidity of the protein, while localized regions displayed expected flexibility consistent with normal protein dynamics. Importantly, no abnormal large-scale conformational instability or unfolding behavior was observed throughout the simulation. Taken together, these findings indicate the structural stability and binding feasibility of DHA with key inflammatory and signaling-related targets, particularly PIK3CA, providing additional mechanistic evidence for the involvement of the PI3K-AKT signaling pathway in DHA-mediated regulation of asthma.

### DHA suppresses HDM induced inflammatory responses in airway epithelial cells via inhibition of PI3K-AKT signaling

**Fig. 4:**
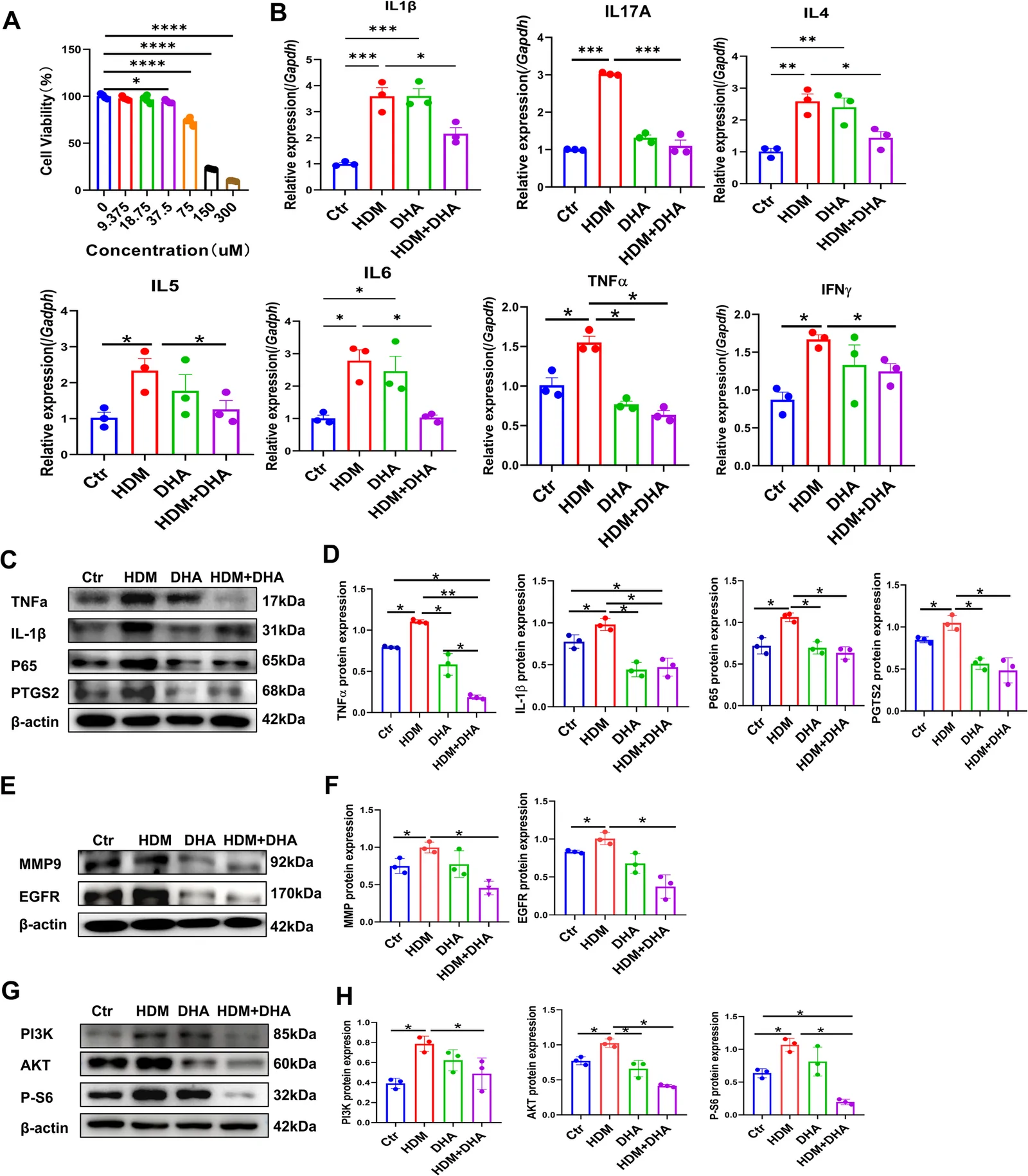
DHA suppresses HDM induced inflammatory responses in HBE cells via inhibition of PI3K-AKT signaling. (**A**) Concentration determination of DHA on HBE cells by CCK8 kit; (**B**) DHA inhibited the mRNA expression of inflammatory cytokines *IL1β*, *IL17A*, *IL4*, *IL5*, *IL6*, *TNFα* and *IFNγ* in HBE; **C-D**. Western blot analysis of IL1β, TNFα, p65 and PTGS2 expression following DHA treatment (**C**) and relative protein expression levels were quantified by densitometric analysis using ImageJ software (**D**) (*n* = 3). E-F. Western blot analysis of EGFR and MMP9 expression following DHA treatment (**E**) and relative protein expression levels were quantified by densitometric analysis using ImageJ software (**F**) (*n* = 3); **G-H**. Western blot revealed that DHA improved the inflammatory response of HBE by affecting the PI3K-AKT signaling pathway (PI3K, AKT, P-S6) and relative protein expression levels were quantified by densitometric analysis using ImageJ software (**F**) (*n* = 3). *p < 0.05, **p < 0.01, ***P < 0.001, **** *P* < 0.0001

To evaluate the biosafety of DHA in airway epithelial cells, cytotoxicity was first assessed in human HBE cells using a CCK-8 assay. DHA treatment at concentrations up to 20 µM did not significantly affect cell viability, indicating that this concentration is well tolerated in vitro (Fig. 4A). The anti-inflammatory effects of DHA were subsequently examined in HDM-stimulated HBE cells. HDM challenge markedly increased the mRNA expression of multiple pro-inflammatory and immune-related cytokines, including *TNFα*, *IFNγ*, *IL1β*, *IL4*, *IL5*, *IL6*, and *IL17A*. Notably, DHA treatment significantly suppressed the HDM-induced upregulation of these cytokines at the transcriptional level (Fig. 4B). Consistent with these transcriptional changes, immunoblot analysis demonstrated that HDM stimulation significantly increased the protein expression of TNF-α, IL-1β, NF-κB p65, and PTGS2, whereas DHA treatment markedly reduced their expression (Fig. 4C-D). In parallel, based on PPI network analysis identifying MMP9 and EGFR as key hub targets, their protein expression was further validated. Western blot results showed that HDM exposure significantly elevated MMP9 and EGFR levels, which were substantially decreased following DHA intervention (Fig. 4E-F). Given that KEGG pathway enrichment analysis highlighted the PI3K-AKT signaling pathway as a central regulatory axis, we next evaluated its activation status in HBE cells. HDM stimulation markedly enhanced the expression of PI3K, AKT, and the downstream effector phosphorylated S6 (p-S6), whereas DHA treatment effectively suppressed their activation (Fig. 4G-H). Collectively, these findings indicate that DHA mitigates HDM-induced airway epithelial inflammation by inhibiting pro-inflammatory mediator production and suppressing PI3K-AKT signaling.

### DHA attenuates airway inflammation and leukocyte infiltration in HDM induced asthmatic mice

**Fig. 5:**
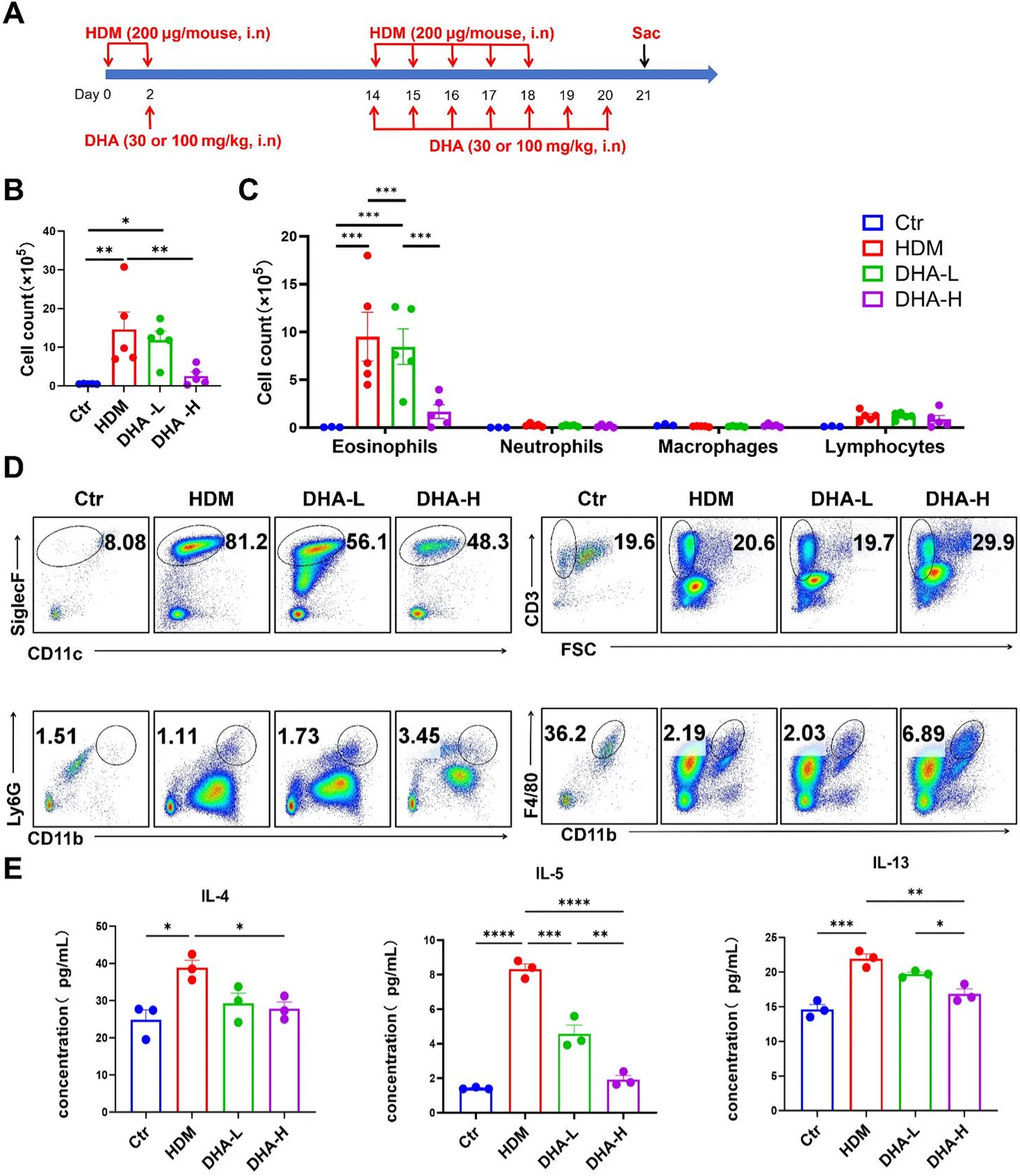
DHA attenuates inflammatory cell infiltration in BALF in an HDM induced asthma model. (**A**) The experimental process of evaluating the anti-asthma effect of DHA in mice, three independent experiments were performed (*n* = 5 per group); (**B**) A leukocyte count of BALF in mice showed that DHA reduced the number of airway leukocytes in asthmatic mice; **C**.DHA reduced the number of eosinophils in the airways of asthmatic mice; **D**. Flow cytometry revealed that DHA reduced the proportion of eosinophils in the airways of asthmatic mice. **E**. ELISA assay showed that DHA could reduce the levels of IL-4, IL-5 and IL-13 in BALF. **p < 0.05, **p < 0.01, ***P < 0.001, **** *P* < 0.0001

To assess the protective effects of DHA against allergic airway inflammation in vivo, an HDM-induced murine asthma model was established, as outlined in Fig. 5A. BALF, lung tissues, and serum samples were collected for analysis following the final HDM challenge. Cytological analysis of BALF revealed a marked increase in total leukocyte counts in HDM challenged mice compared with control animals. DHA treatment significantly reduced total inflammatory cell infiltration into the airways, with the high-dose DHA group exhibiting a more pronounced reduction than the low dose group (Fig. 5B). Differential cell counting further demonstrated that HDM exposure robustly increased eosinophil and neutrophil populations, whereas DHA administration significantly decreased these inflammatory cell subsets in a dose dependent manner (Fig. 5C). Consistent with these findings, flow cytometric analysis confirmed substantial reductions in airway-infiltrating eosinophils and neutrophils in DHA-treated mice. The inhibitory effect was more prominent in the high dose DHA group, indicating a clear dose-dependent suppression of HDM-induced airway inflammation (Fig. 5D). In parallel, ELISA of BALF demonstrated that levels of Th2-associated cytokines, including IL-4, IL-5, and IL-13, were significantly elevated in HDM-challenged mice but markedly reduced following DHA treatment (Fig. 5E). In addition, consistent results across three independent experiments further supported the robustness and reproducibility of our findings. Collectively, these results indicate that DHA effectively alleviates allergic airway inflammation by suppressing leukocyte recruitment and Th2 type inflammatory responses in vivo.

### DHA modulates pulmonary inflammation in asthmatic mice

**Fig. 6:**
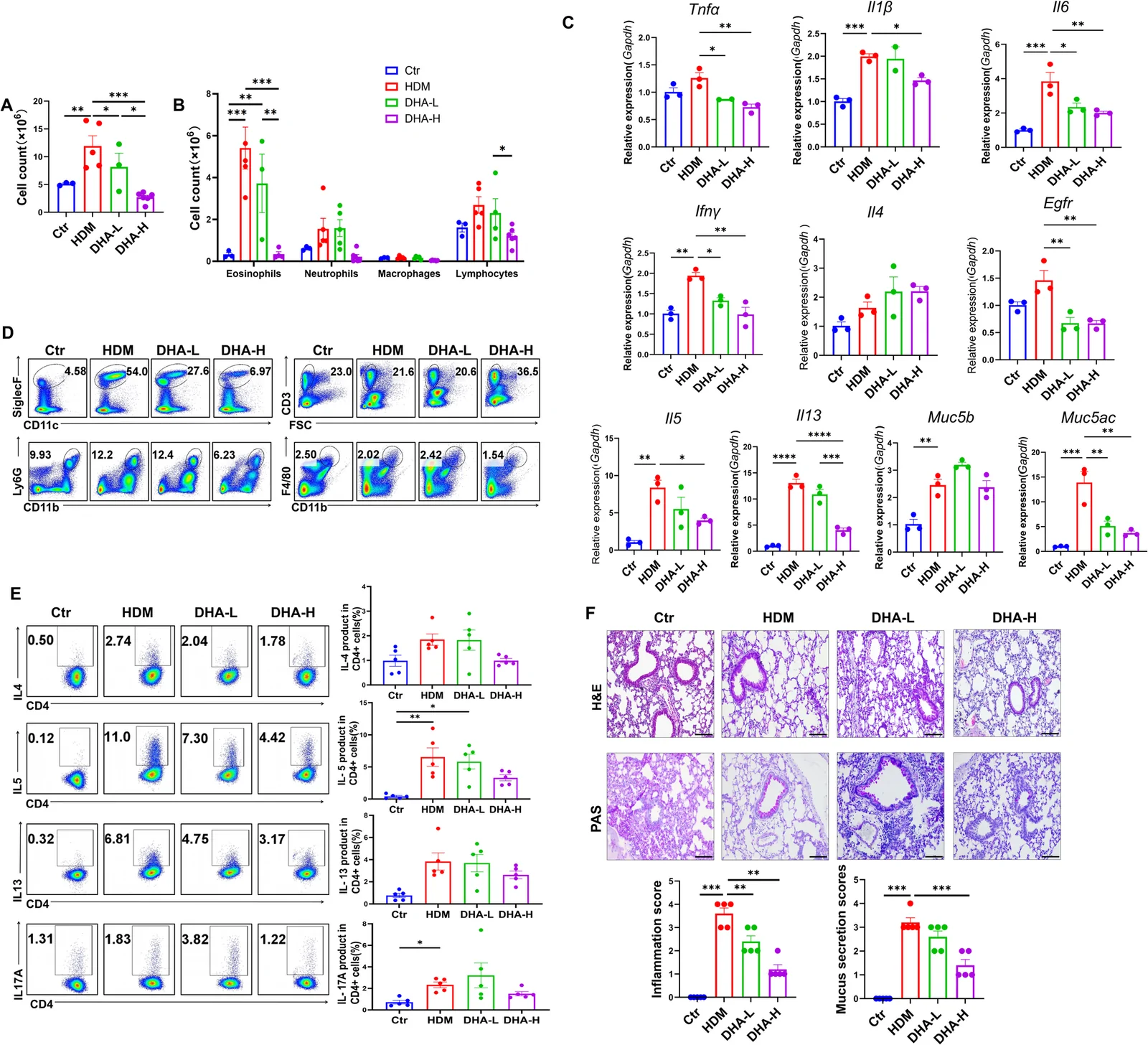
DHA modulates Th2 skewed immune responses in vivo. **A**. Cell count showed that DHA reduced the number of white blood cells in the lungs of asthmatic mice; **B**. DHA decreased the quantity of inflammatory cells in the lungs of asthmatic mice; **C**. DHA lowered the mRNA expression levels of inflammatory factors in asthmatic mice; **D**. Flow cytometry found that DHA reduced the proportion of inflammatory cells in the lungs of asthmatic mice; **E**. Flow cytometry showed that DHA reduced the expression of inflammatory cytokines IL-4, IL-5, IL-13 and IL-17 A in the lungs of asthmatic mice; **F**. Histopathological sections of mouse lung showed that H&E and PAS staining showed that inflammatory cell infiltration and high mucus secretion were reduced in mice treated with DHA, and the lung tissue structure was almost completely restored in the high dose group. **G**. The pathological scores of H&E and PAS staining in lung tissue of mice in high-dose DHA group decreased significantly. *p < 0.05, **p < 0.01, ***P < 0.001, **** P < 0.0001

We next evaluated the in vivo impact of DHA on airway inflammatory responses and related signaling activity in HDM-induced asthmatic mice. Immunoblotting of lung tissues indicated that DHA treatment was associated with attenuation of inflammatory signaling. Consistent with this observation, total leukocyte counts were significantly decreased in both low- and high-dose DHA groups compared with the HDM model group, with the high-dose regimen producing the most pronounced reduction (Fig. 6A). Differential cell analysis further demonstrated that DHA markedly suppressed the recruitment of major inflammatory subsets, particularly eosinophils and neutrophils, in a dose-dependent manner (Fig. 6B). At the transcriptional level, RT-qPCR confirmed an overall downregulation of pro-inflammatory cytokine mRNA expression in lung tissues following DHA treatment (Fig. 6C).

To further characterize the immunomodulatory characteristics of DHA, we found by flow cytometry that HDM challenged mice exhibited significant upregulation of lung eosinophils compared with normal controls. Notably, high-dose DHA significantly increased the proportion of lung eosinophils (Fig. 6D). Flow cytometric analysis further revealed that HDM exposure markedly increased the proportions of IL-4, IL-5, and IL-13-producing Th2 cells, as well as IL-17-producing Th17 cells, in lung tissues compared with normal mice. DHA treatment, particularly at the high dose, significantly reduced Th2 cell frequencies, whereas the proportion of IL-17⁺ Th17 cells showed no statistically significant change among groups (Fig. 6E). Histopathological examination using H&E staining confirmed that DHA administration markedly alleviated leukocyte infiltration and peribronchial inflammatory accumulation in the lungs of HDM-challenged mice (Fig. 6F). The inflammation scores of lung tissue pathological sections of mice in each group were evaluated. The results showed that compared with HDM group mice, the pathological scores of H&E and PAS staining of lung tissue pathological sections of mice treated with DHA were reduced, and the pathological scores of H&E and PAS staining of lung tissue of mice in high-dose DHA group were lower(Fig. 6G). Collectively, these findings demonstrate that DHA effectively mitigates allergen-induced airway inflammation in vivo.

### DHA inhibits PI3K-AKT-mTOR signaling in the asthmatic lung

**Fig. 7:**
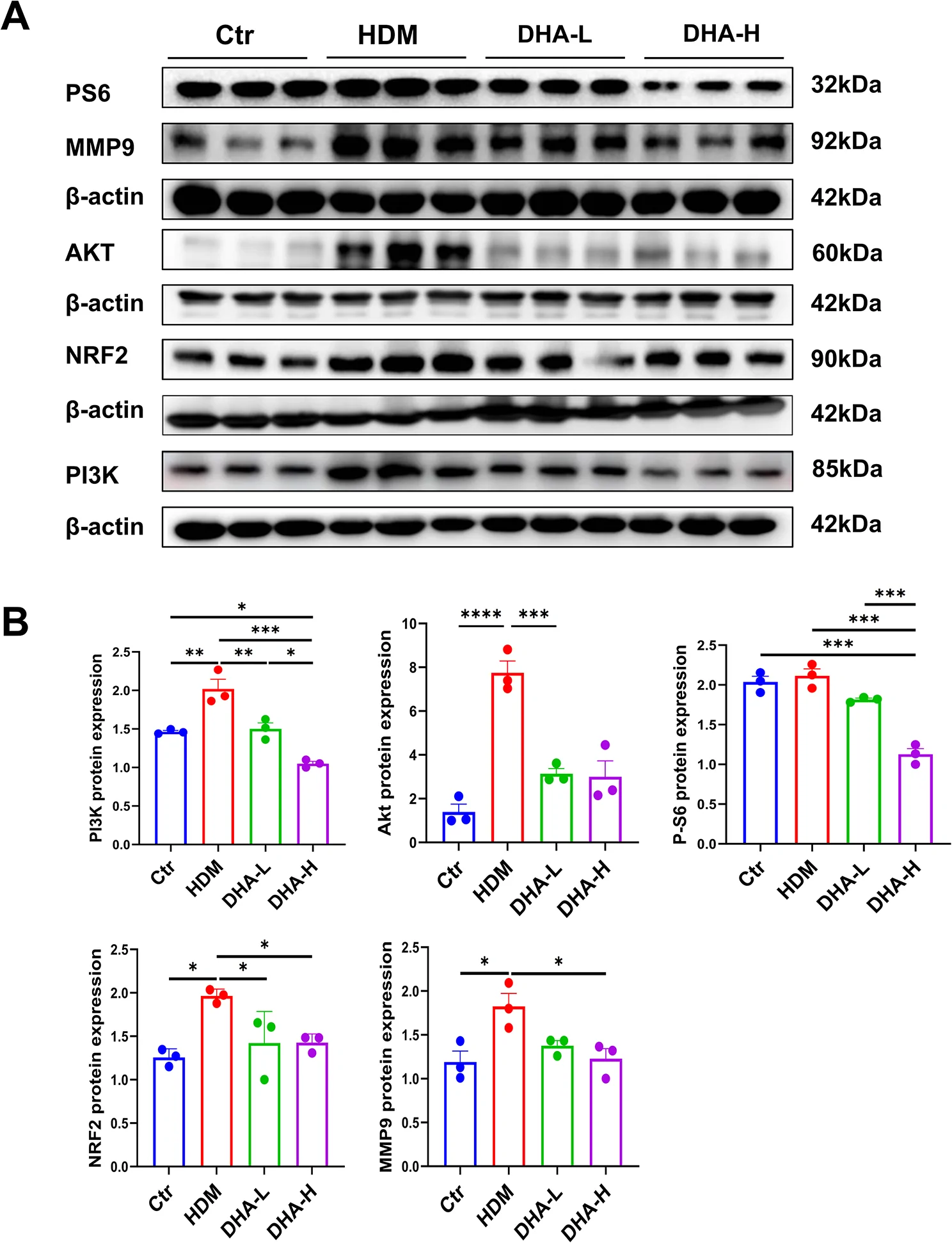
DHA inhibits PI3K-AKT-mTOR signaling in the asthmatic lung. (**A**) Immunoprotein blot results showed that DHA improved the pathological process by acting on the PI3K-AKT-mTOR signaling pathway in the lungs of asthmatic mice (Three specimens were randomly selected; *n* = 3); (**B**) Use ImageJ software to quantify the relative expression of each band in the Western blot. *p < 0.05, **p < 0.01, ***P < 0.001, **** *P* < 0.0001

To elucidate the molecular mechanisms underlying the protective effects of DHA in allergic asthma, we examined activation of the PI3K-AKT signaling pathway in lung tissues from HDM-induced asthmatic mice. Network pharmacology analysis and KEGG pathway enrichment had previously identified the PI3K-AKT axis as a potential key pathway targeted by DHA. Consistent with these predictions, immunoblot analysis showed that HDM exposure markedly activated the PI3K-AKT-mTOR signaling cascade, as indicated by increased expression of PI3K, AKT, mTOR, NRF2, and the downstream effector phosphorylated ribosomal protein S6 (p-S6) (Fig. 7A-B).

DHA treatment significantly suppressed activation of this pathway. Compared with the HDM model group, DHA markedly reduced the protein levels of PI3K, AKT, mTOR, NRF2, and p-S6 in lung tissues, with the high-dose DHA group showing the strongest inhibitory effect. Given that the PI3K-AKT-mTOR pathway plays a central role in regulating airway inflammation, oxidative stress, and airway remodeling, its suppression by DHA likely represents an important mechanism underlying the attenuation of allergic airway inflammation. Overall, these results, together with the network pharmacology predictions, indicate that DHA alleviates HDM induced allergic airway inflammation at least partly through inhibition of the PI3K-AKT-mTOR signaling pathway.

## Discussion

Asthma is a heterogeneous chronic airway disease characterized by persistent inflammation, airway hyperresponsiveness, and progressive remodeling, driven by complex interactions among immune and structural cells within the airway microenvironment [32, 33]. Despite substantial improvements in symptom control with inhaled corticosteroids and biologics, these therapies are not curative and are often limited by variable responsiveness, steroid resistance, and adverse effects [34, 35]. Therefore, identifying therapeutic agents capable of modulating upstream inflammatory networks rather than single mediators is of considerable clinical significance.

DHA, the principal metabolite of *artemisinin*, has gained attention for its broad anti-inflammatory and immunomodulatory properties in addition to its antimalarial activity [36, 37]. Previous studies have shown that DHA significantly reduces inflammatory cell infiltration and decreases OVA-specific IgE levels in experimental asthma models [38]. In addition, DHA has been reported to suppress the expression of mucus-associated genes, including MUC5AC and chitinase 3-like protein 4 (YM2), in OVA-induced lung inflammation. Consistent with these findings, artemisinin derivatives have been demonstrated to ameliorate experimental allergic airway inflammation, at least in part, through negative regulation of the PI3K/AKT signaling pathway and its downstream NF-κB activity. In these studies, treatment with artemisinin derivatives dose-dependently reduced OVA-induced increases in total eosinophil counts as well as the levels of IL-4, IL-5, IL-13, and eosinophil chemokines in BALF [39]. Similarly, it has been reported that artemisinin derivatives attenuate oxidative stress in OVA challenged mouse lung tissue and TNF-α stimulated human bronchial epithelial cells, as evidenced by decreased levels of 8-isoprostane, 8-hydroxy-2-deoxyguanosine, and 3-nitrotyrosine, alongside enhanced nuclear translocation of Nrf2 [40]. Mechanistically, DHA has been reported to rebalance immune responses by suppressing mTOR signaling, inhibiting Th1/Th17 differentiation, and promoting Treg expansion via the TGF-β/Smad pathway, without inducing global immunosuppression [10, 41]. Its efficacy has been demonstrated in autoimmune diseases such as SLE through Treg/Th17 modulation [22, 42], in cancers via PI3K/AKT inhibition, and in inflammatory injuries through suppression of 5-LOX, COX-2, and ROS-JNK1/2-autophagy pathways [43, 44]. However, the precise mechanisms of DHA in allergic asthma have remained incompletely defined.

In this study, the combination of network pharmacology and experimental validation enabled systematic elucidation of DHA’s anti-asthmatic mechanisms. Network analysis identified 68 DHA-associated asthma targets, with hub genes including EGFR, MAPK3, PTGS2, MMP9, MAPK1, ERBB2, PARP1, MAP2K1, and JAK2. Functional enrichment highlighted receptor-mediated inflammatory signaling, with the PI3K-AKT pathway emerging as a central regulatory axis. Molecular docking supported direct interaction between DHA and PI3K catalytic subunits, suggesting PI3K-AKT as a primary molecular target. Dysregulated PI3K-AKT signaling is known to drive NF-κB dependent proinflammatory cytokine expression, Th2 polarization, epithelial barrier dysfunction, and steroid insensitivity [45, 46], collectively linking immune dysregulation, epithelial pathology, and chronic airway inflammation [47]. Consistent with computational predictions, in vivo and in vitro studies demonstrated that DHA significantly attenuated HDM induced airway inflammation. DHA reduced Th2 cytokines (IL-5, IL-13), proinflammatory mediators (IL-1β, IL-6, TNF-α, IFN-γ, IL-17 A), eosinophil infiltration, and total inflammatory cell counts in bronchoalveolar lavage fluid. Mechanistically, DHA inhibited PI3K-AKT activation and downstream phosphorylation of S6, and downregulated EGFR, MMP9, PTGS2, and NF-κB, confirming suppression of both signaling and effector pathways involved in airway inflammation and remodeling. The strong concordance between network pharmacology predictions and experimental validation underscores the robustness of this integrated approach: PI3K-AKT not only serves as a central signaling hub, but also mediates broader inflammatory networks targeted by DHA.

Despite these advances, several limitations of the present study should be acknowledged. First, the cell type specific regulatory effects of DHA on PI3K-AKT signaling across distinct immune and structural cell populations remain unclear, and warrant further investigation. Second, asthma is a highly heterogeneous disease, encompassing eosinophilic, neutrophilic, mixed granulocytic, and oligogranulocytic phenotypes. However, the current study established the allergic airway disease model using only male mice, without inclusion of female animals. This limits the ability to evaluate potential sex-dependent differences in the anti-inflammatory effects of DHA and its regulation of related signaling pathways. Future studies incorporating both male and female animals are therefore required to further clarify the mechanistic specificity and potential sex-related variability of DHA. Finally, within the framework of precision medicine, the therapeutic efficacy and personalized applicability of DHA across different asthma endotypes remain to be systematically investigated.

## Conclusion

This study demonstrates that DHA alleviates allergic airway inflammation through coordinated inhibition of the PI3K-AKT signaling pathway. By integrating network pharmacology with in vivo and in vitro validation, we identify PI3K-AKT as a central regulatory axis linking epithelial inflammatory activation and immune dysregulation in allergic asthma. Suppression of this pathway by DHA is associated with reduced expression of key downstream mediators, including EGFR, MMP9, and PTGS2, leading to attenuation of inflammatory cell recruitment, type 2 cytokine production, and mucus hypersecretion. These findings extend current understanding of the anti-inflammatory mechanisms of DHA and highlight PI3K-AKT signaling as a nodal pathway amenable to therapeutic modulation in asthma. Together, our results provide a mechanistic basis for further exploration of DHA as a network-oriented immunomodulatory agent and support the rationale for targeting PI3K-AKT centered signaling networks in the treatment of allergic airway diseases.

## Supplementary Information

Below is the link to the electronic supplementary material.

Supplementary Material 1

## Data Availability

The data used to support the findings of this study are included within the article; The raw data will be made available on request.
