# Supplementary material for: Dihydroartemisinin alleviates allergic airway inflammation by suppressing the PI3K-AKT signaling axis: integrated network pharmacology and experimental validation
Source: Respir Res. 2026 Oct 15;27:427. doi: 10.1186/s12931-026-03956-0 (PMC13723100; doi:10.1186/s12931-026-03956-0)

Dihydroartemisinin alleviates allergic airway inflammation by suppressing the PI3K-AKT signaling axis: integrated network pharmacology and experimental validation

Original image for Western blot

fig.4C-original(1)

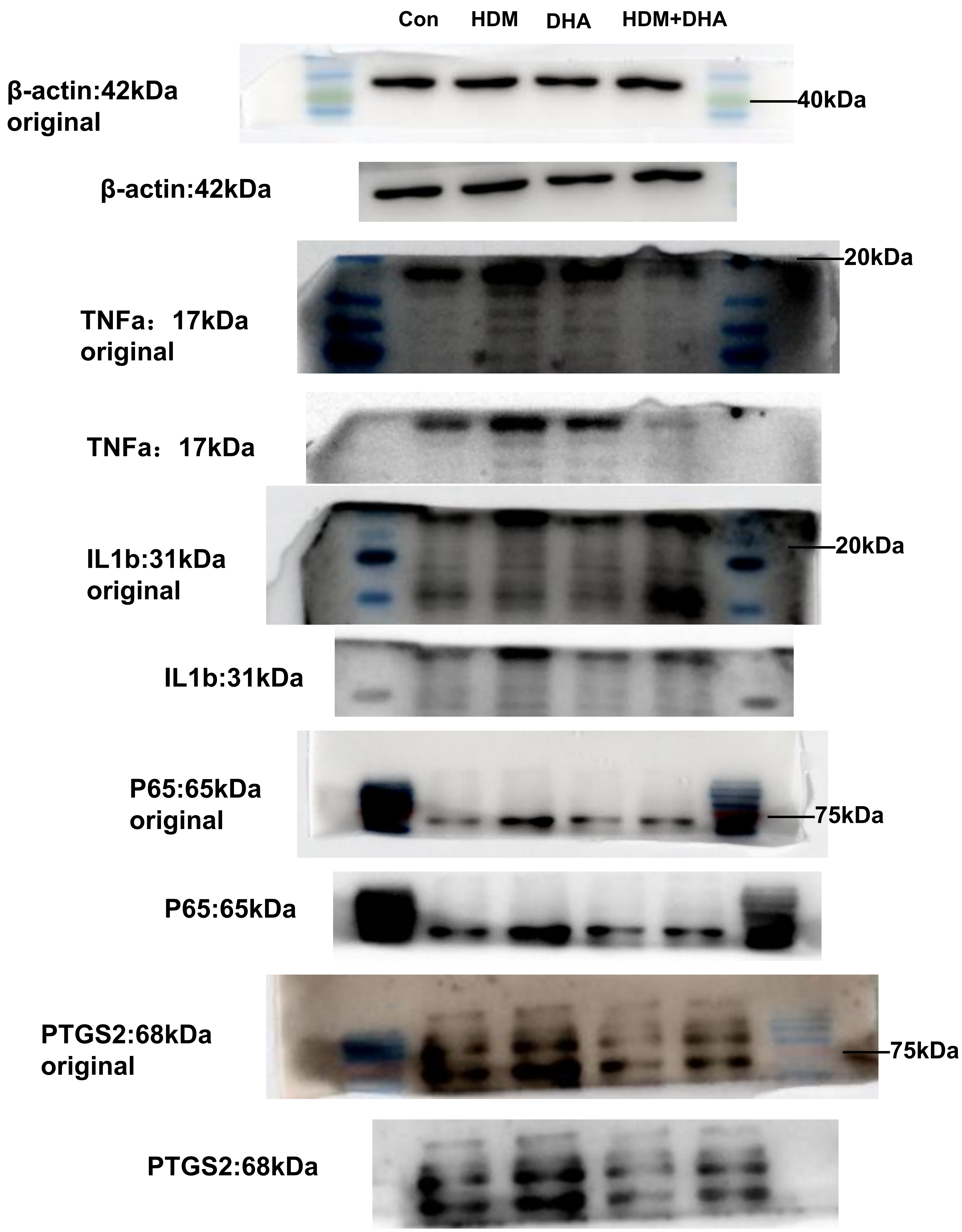

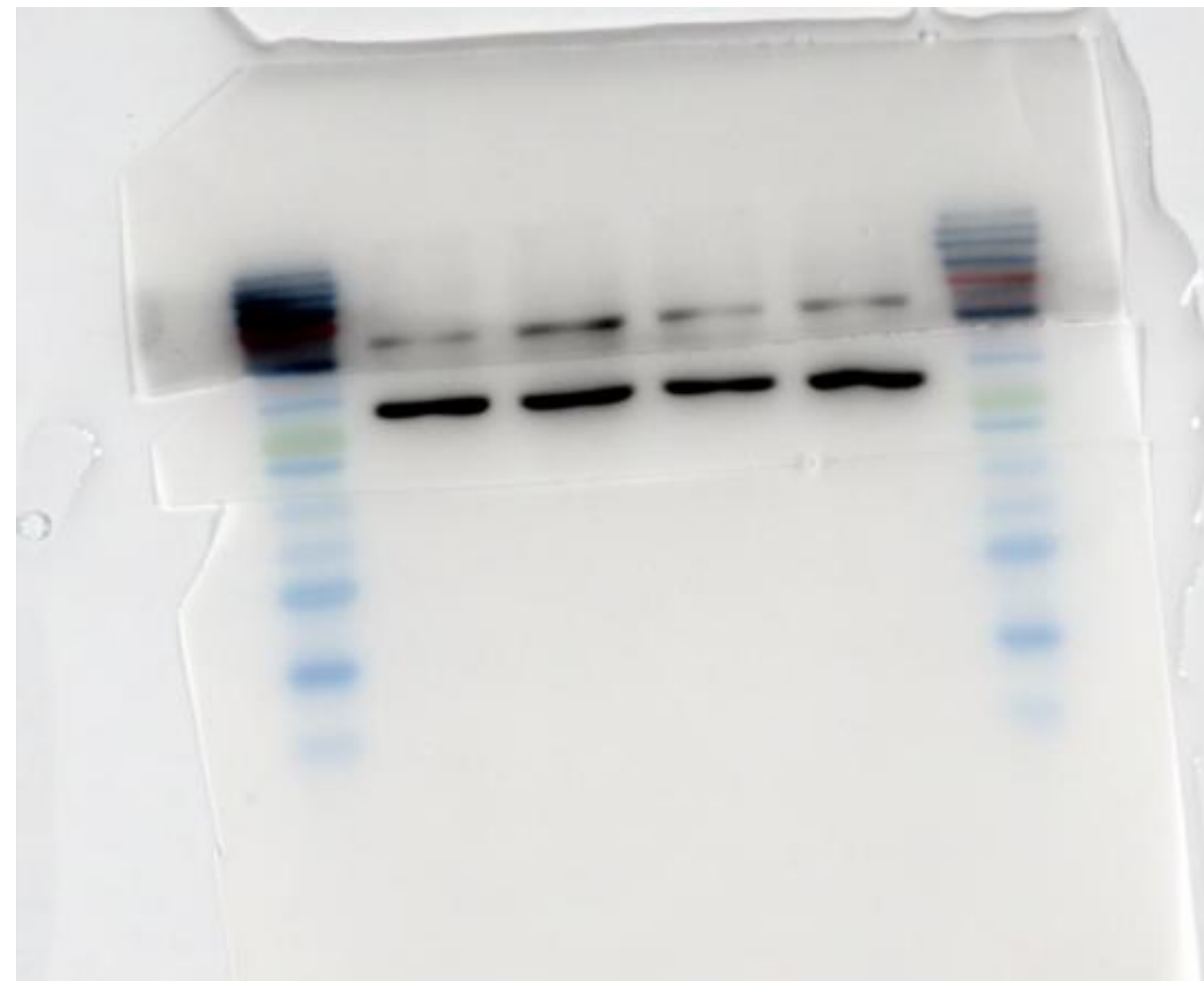

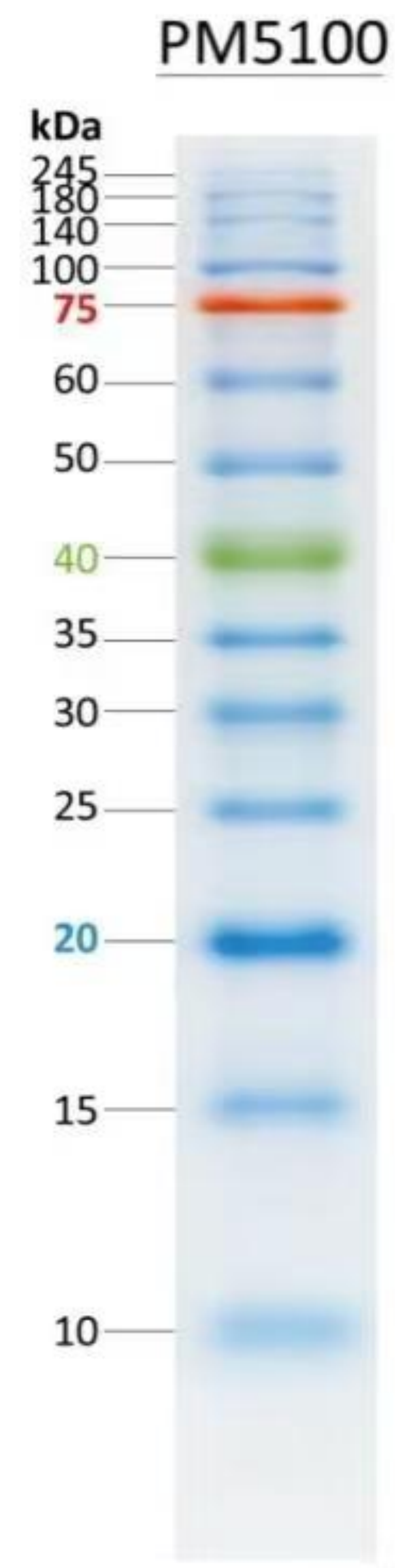

fig.4C-original(2)

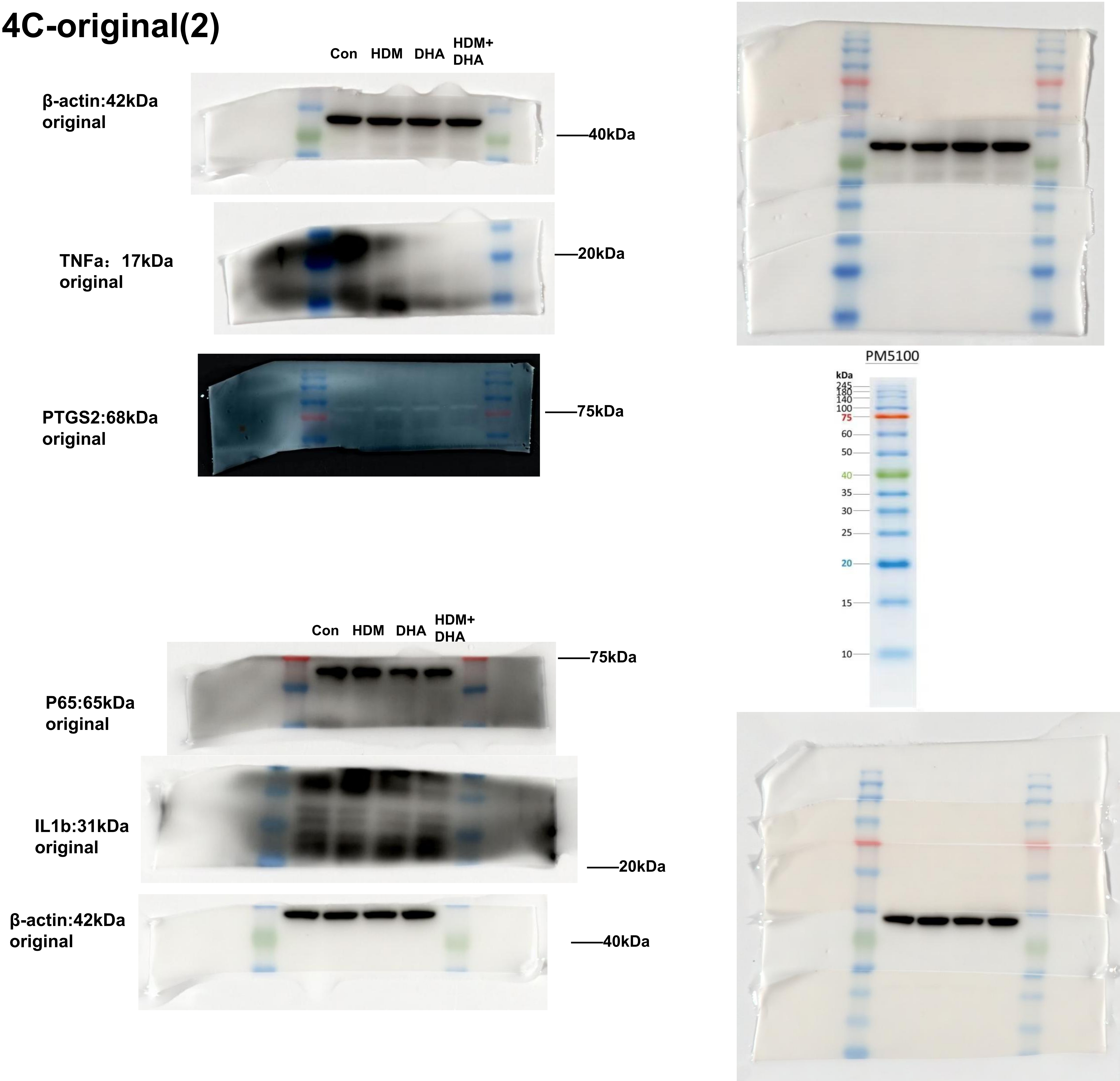

fig.4C-original(3)

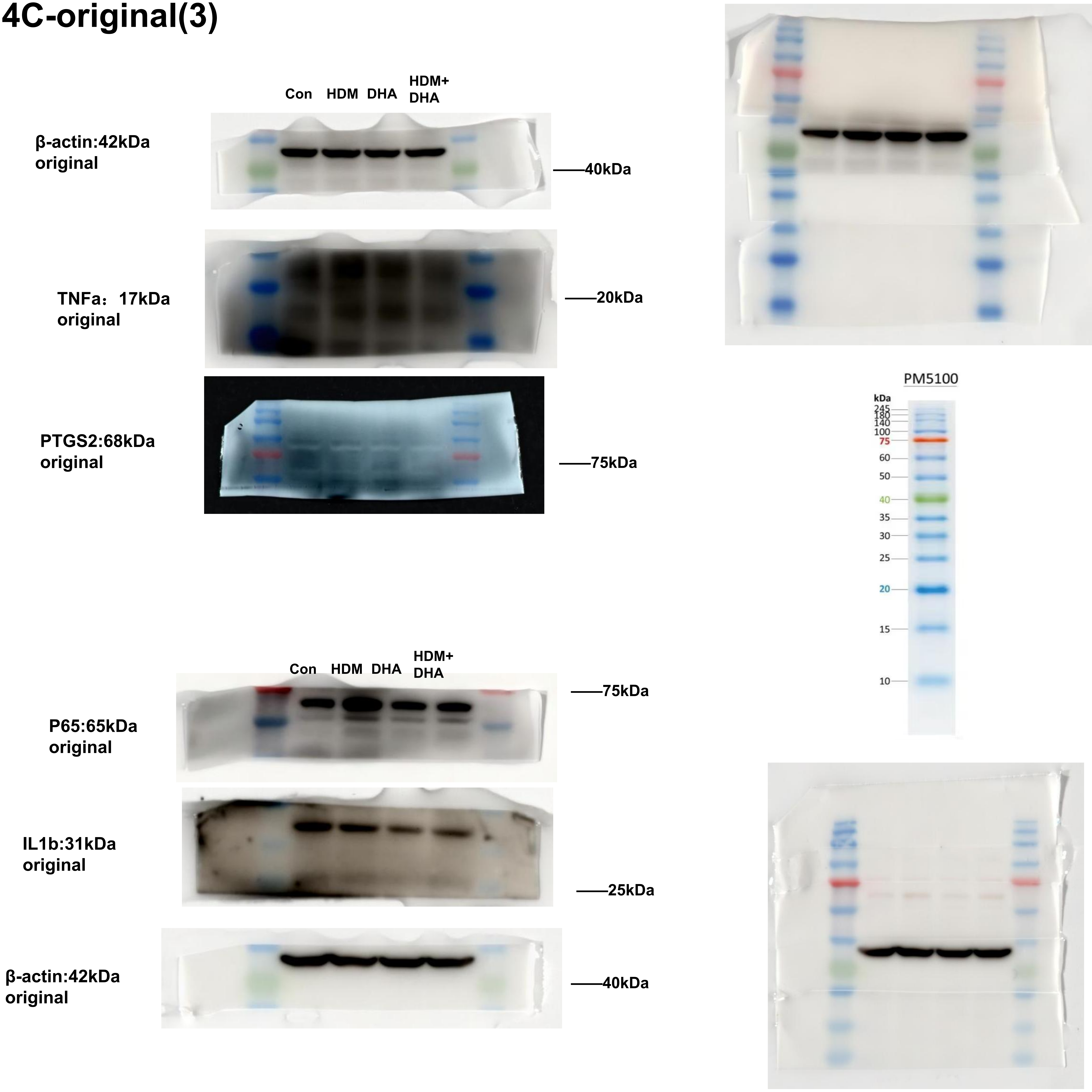

fig.4E-original(1)

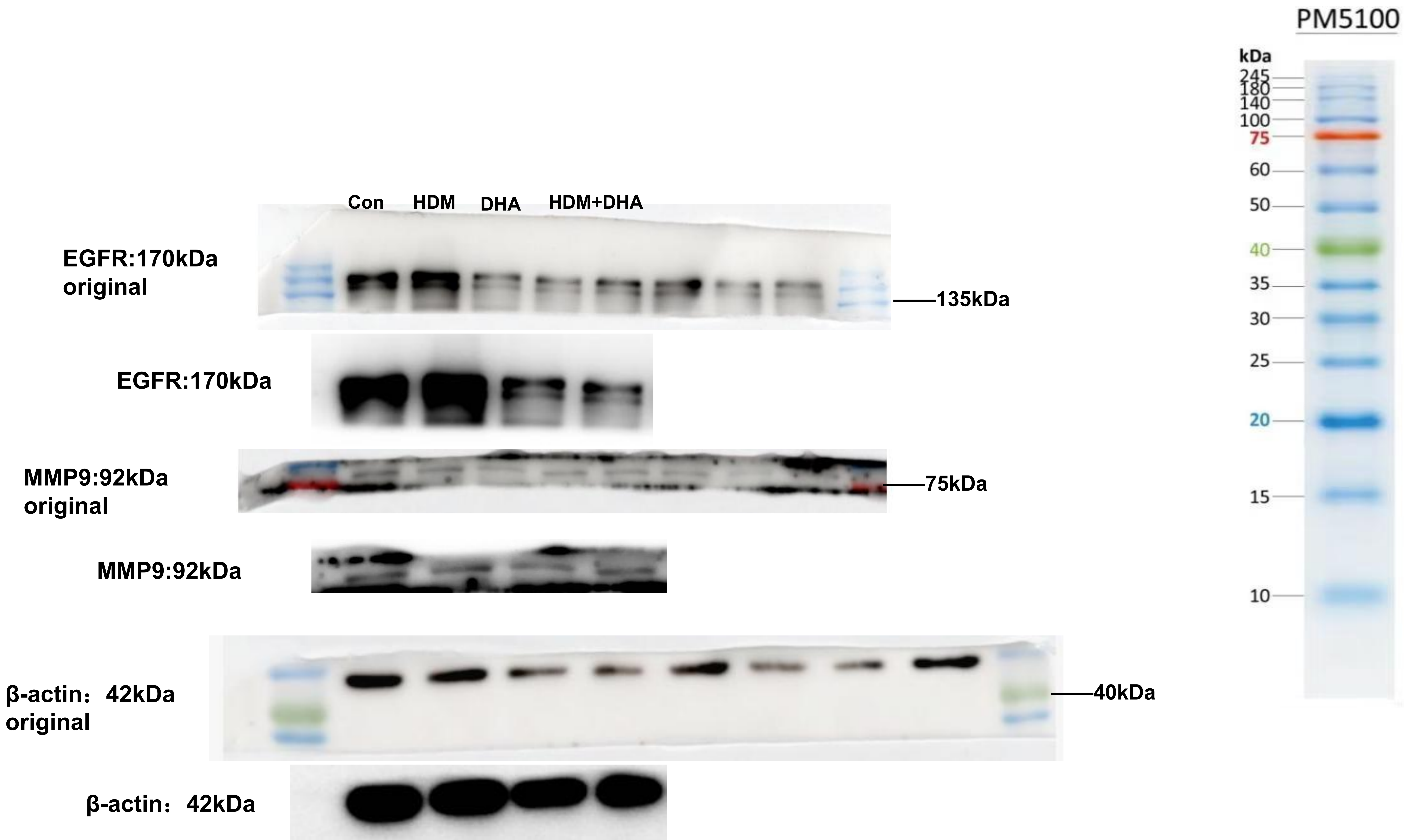

fig.4E-original(2)

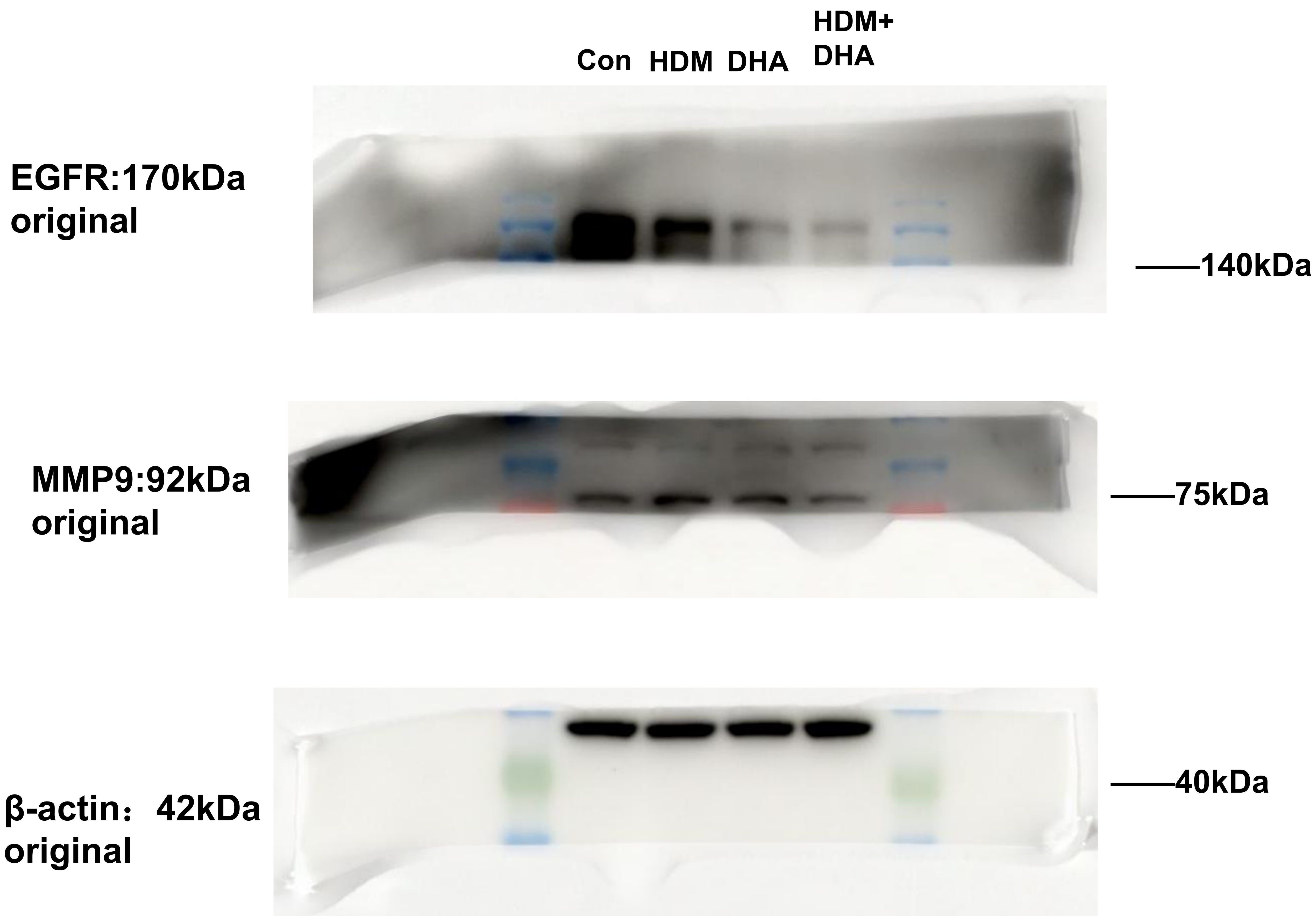

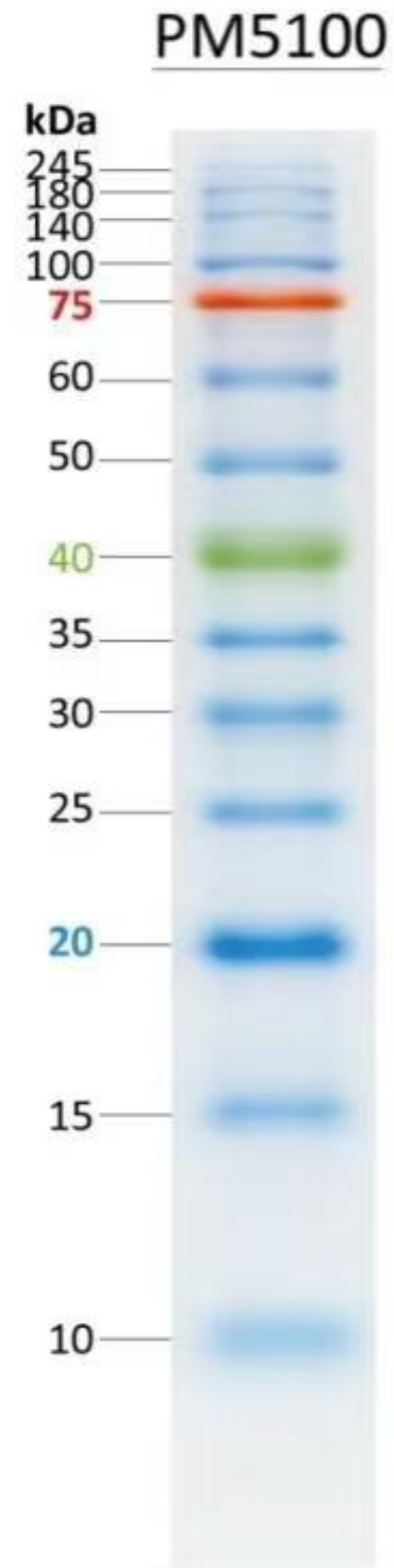

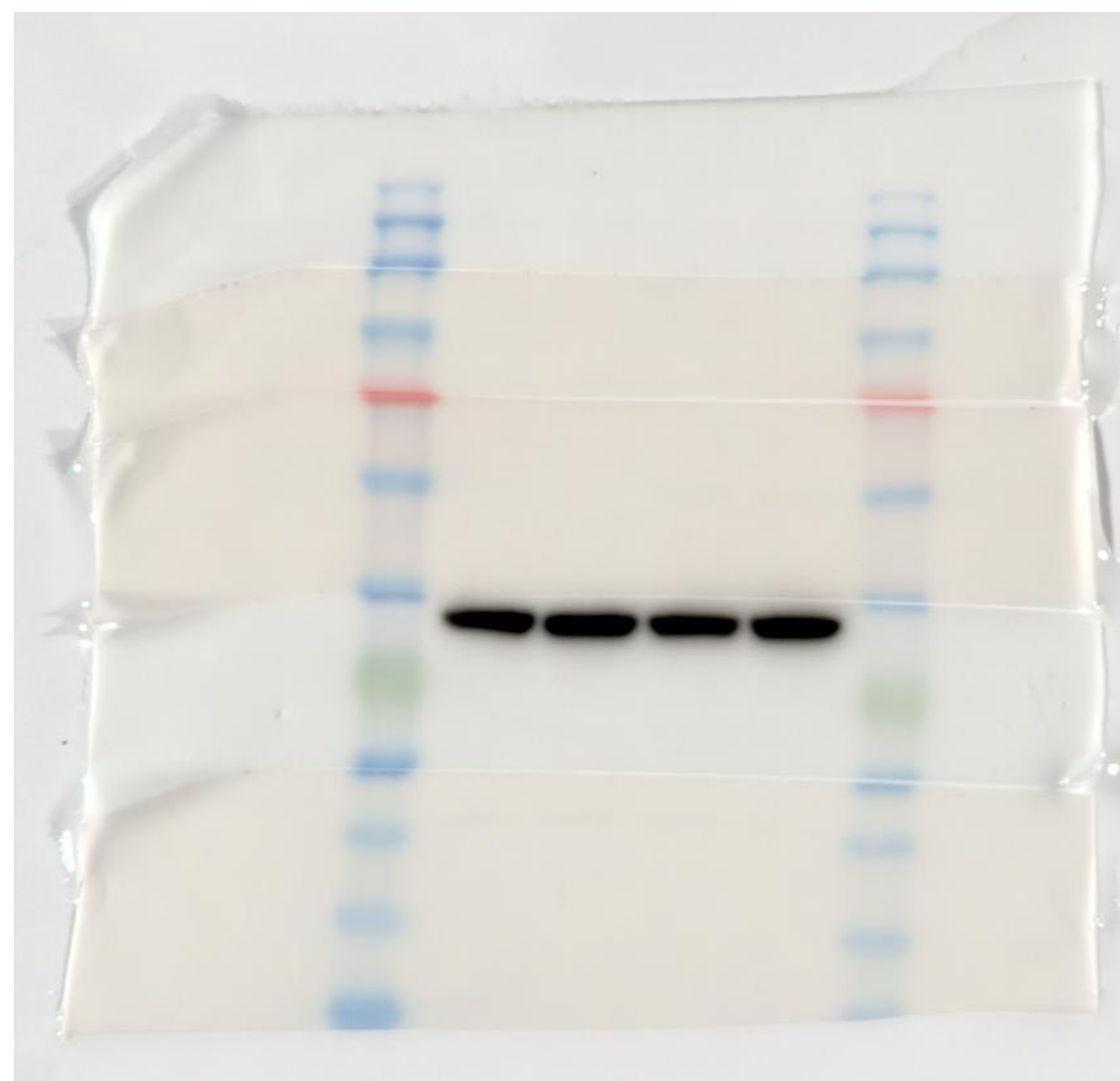

fig.4E-original(3)

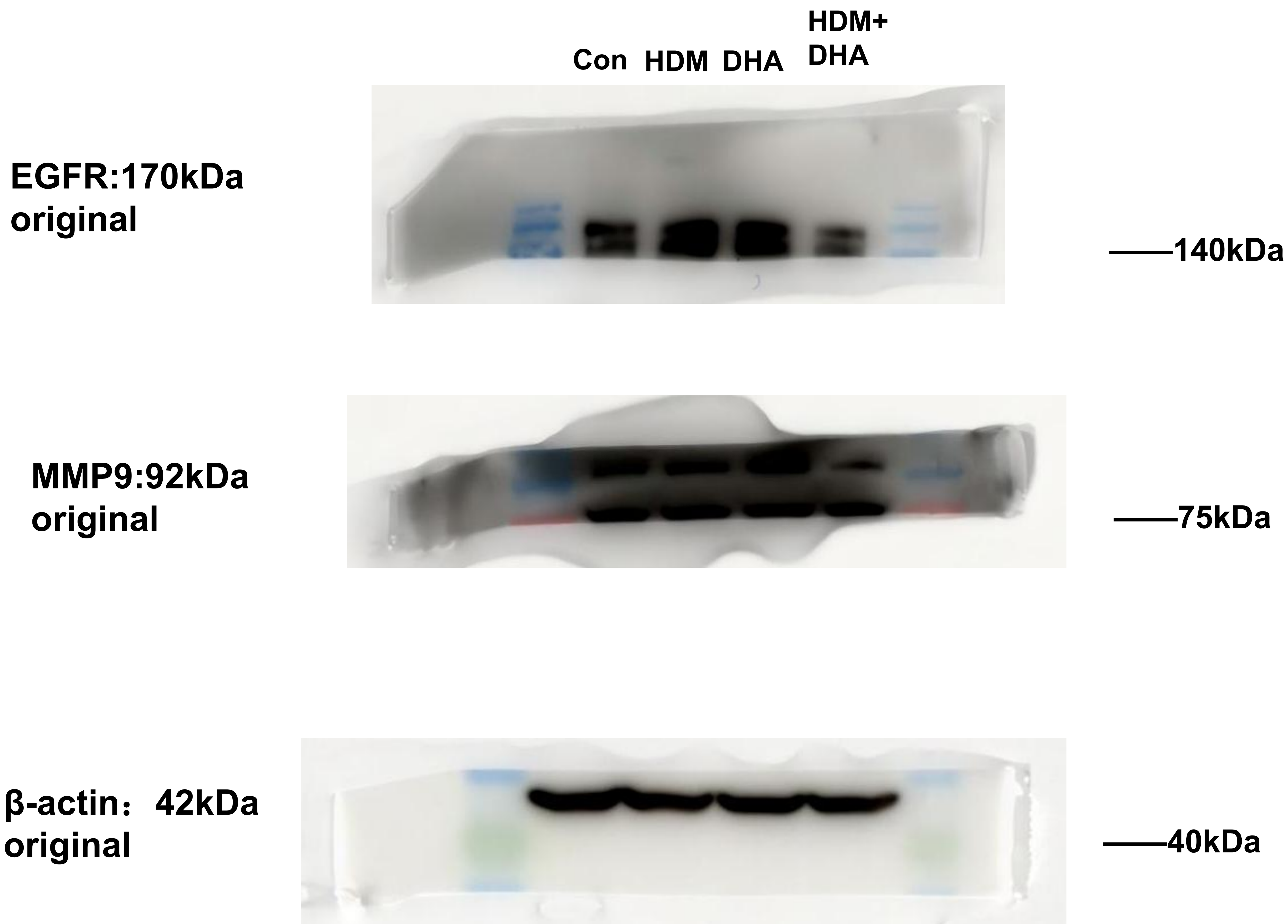

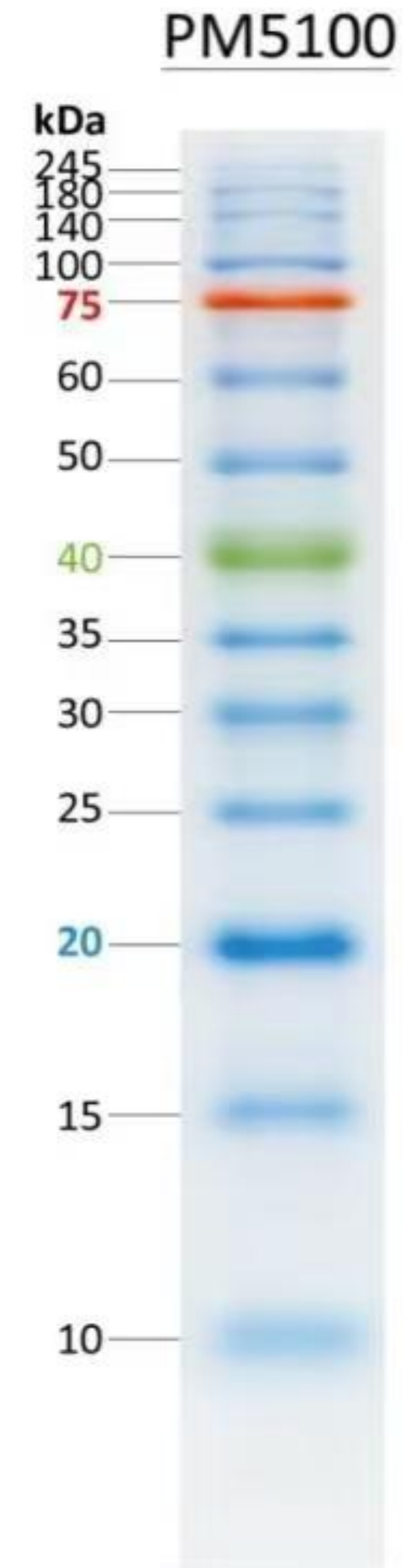

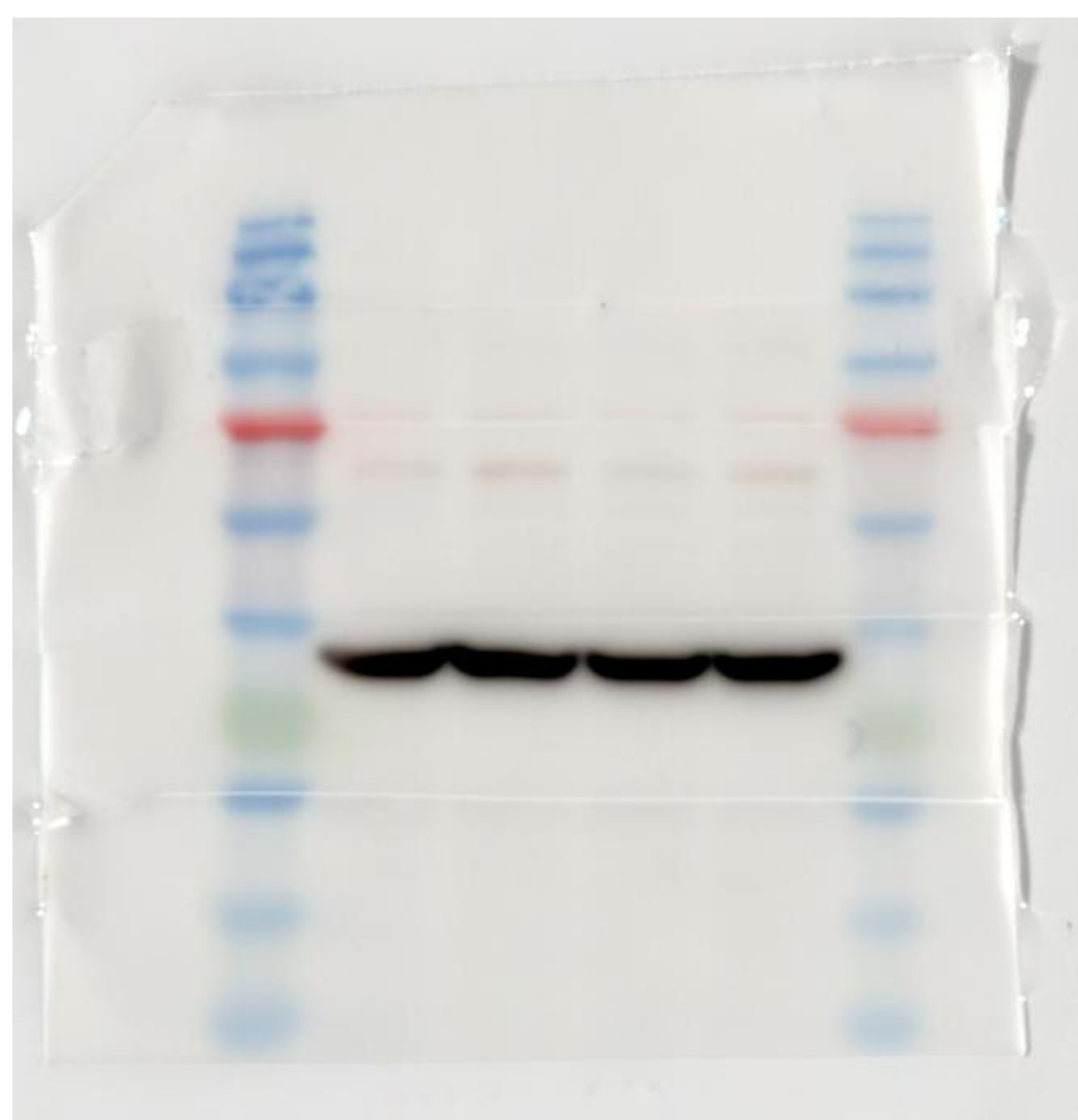

fig.4G-original(1)

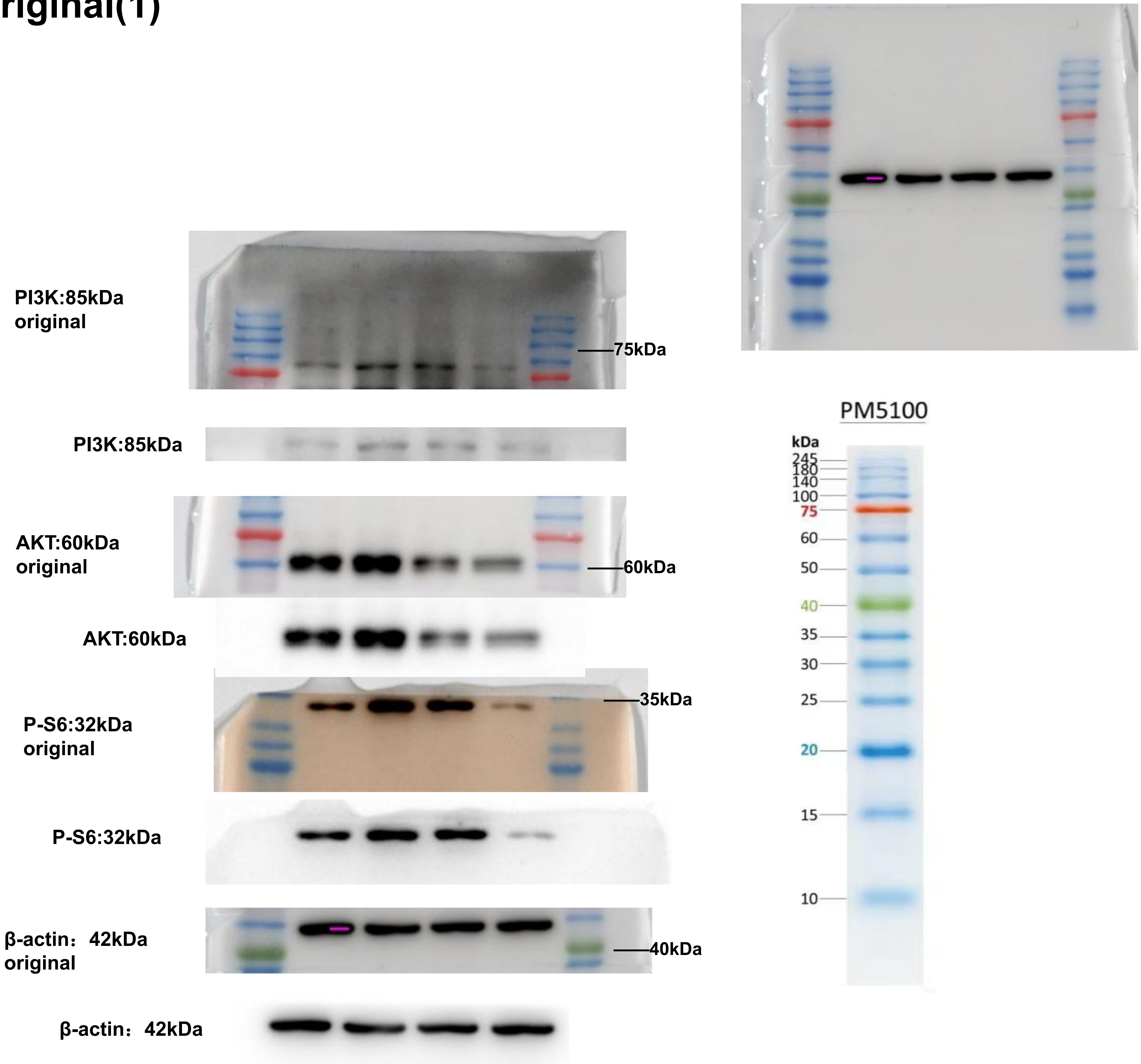

fig.4G-original(2)

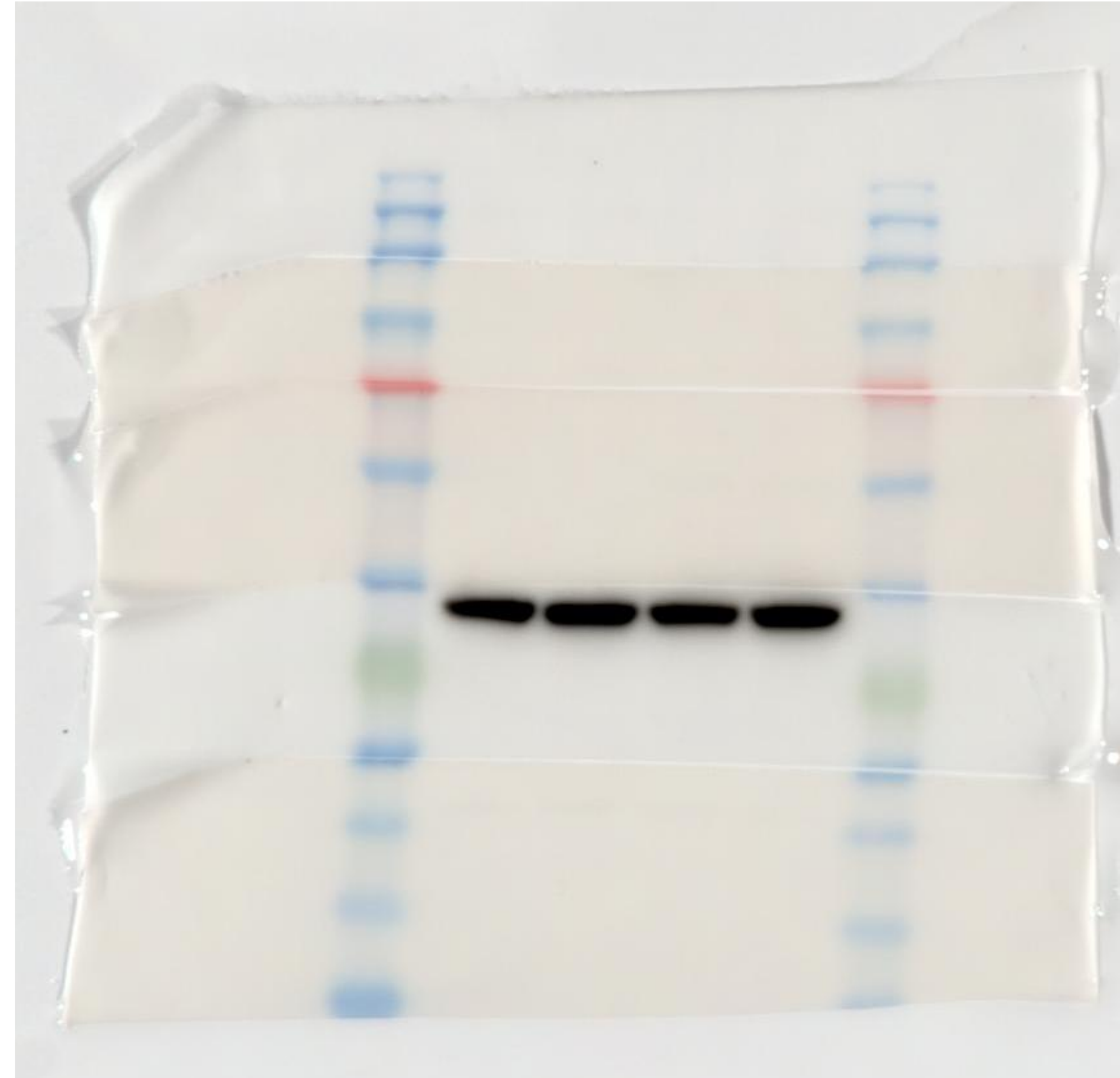

PI3K:85kDa
original

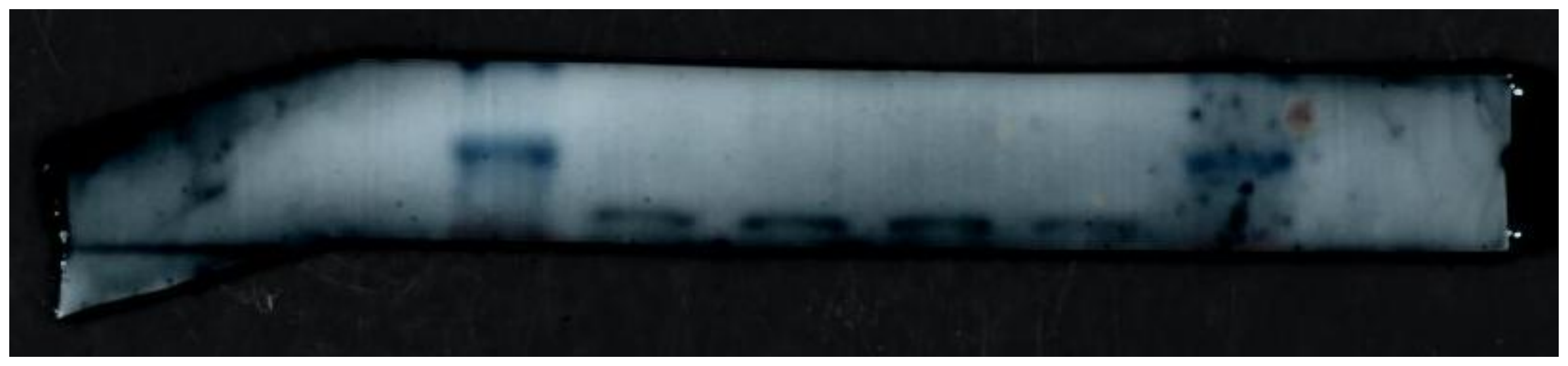

—75kDa

AKT:60kDa
original

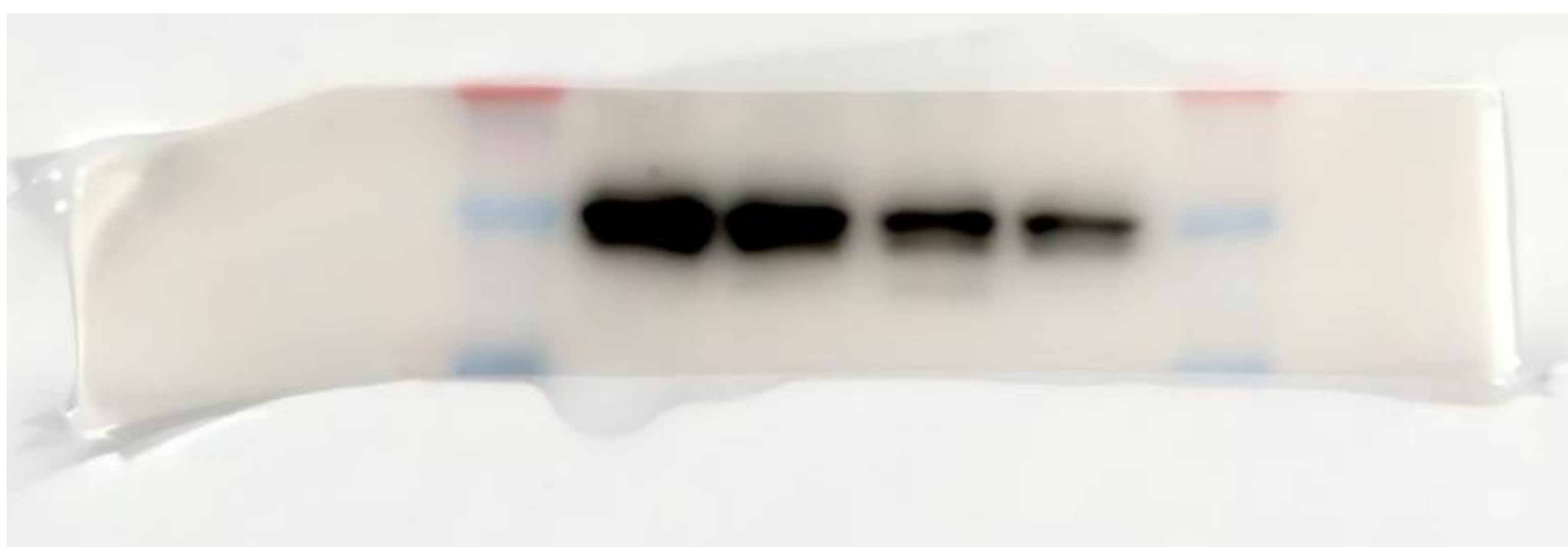

—60kDa

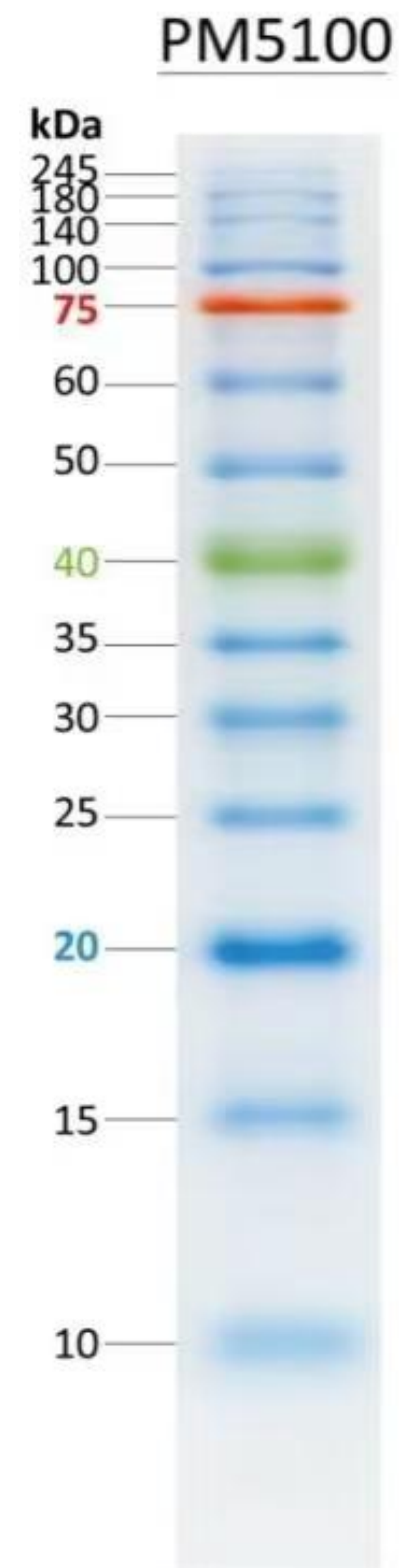

—35kDa

P-S6:32kDa
original

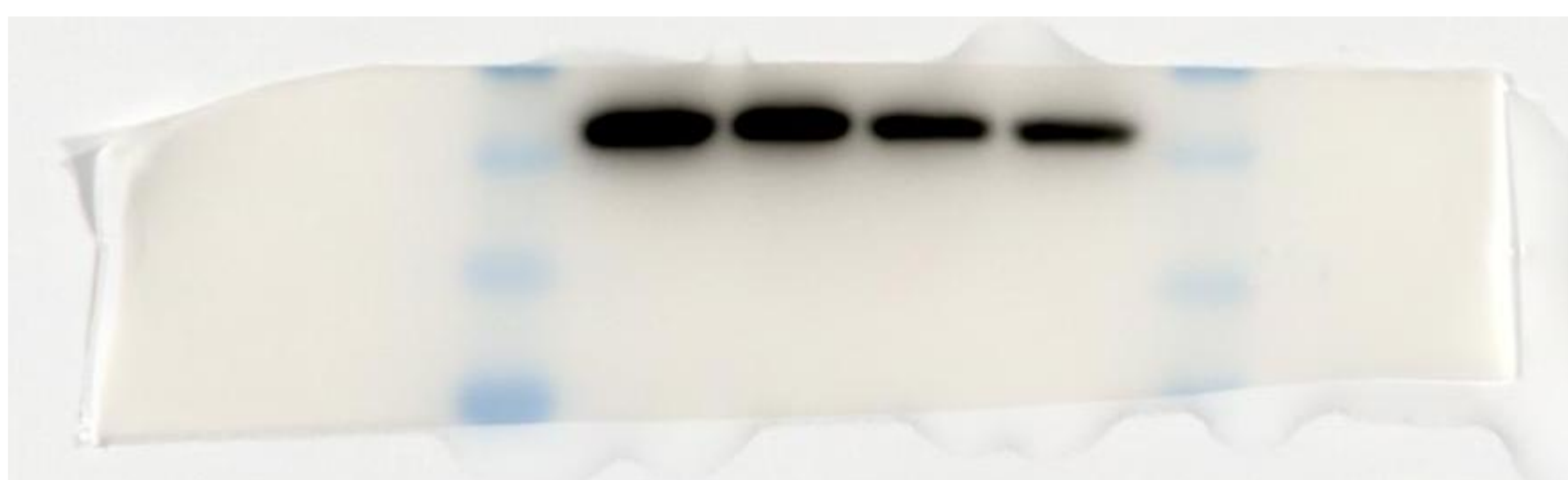

β-actin：42k
original

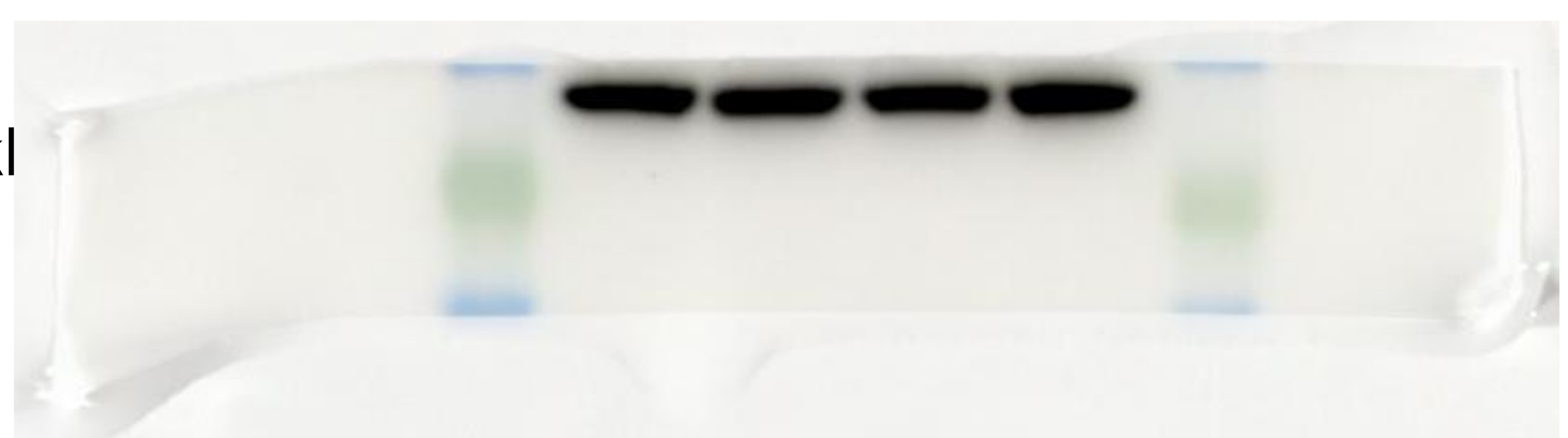

—40kDa

fig.4G-original(3)

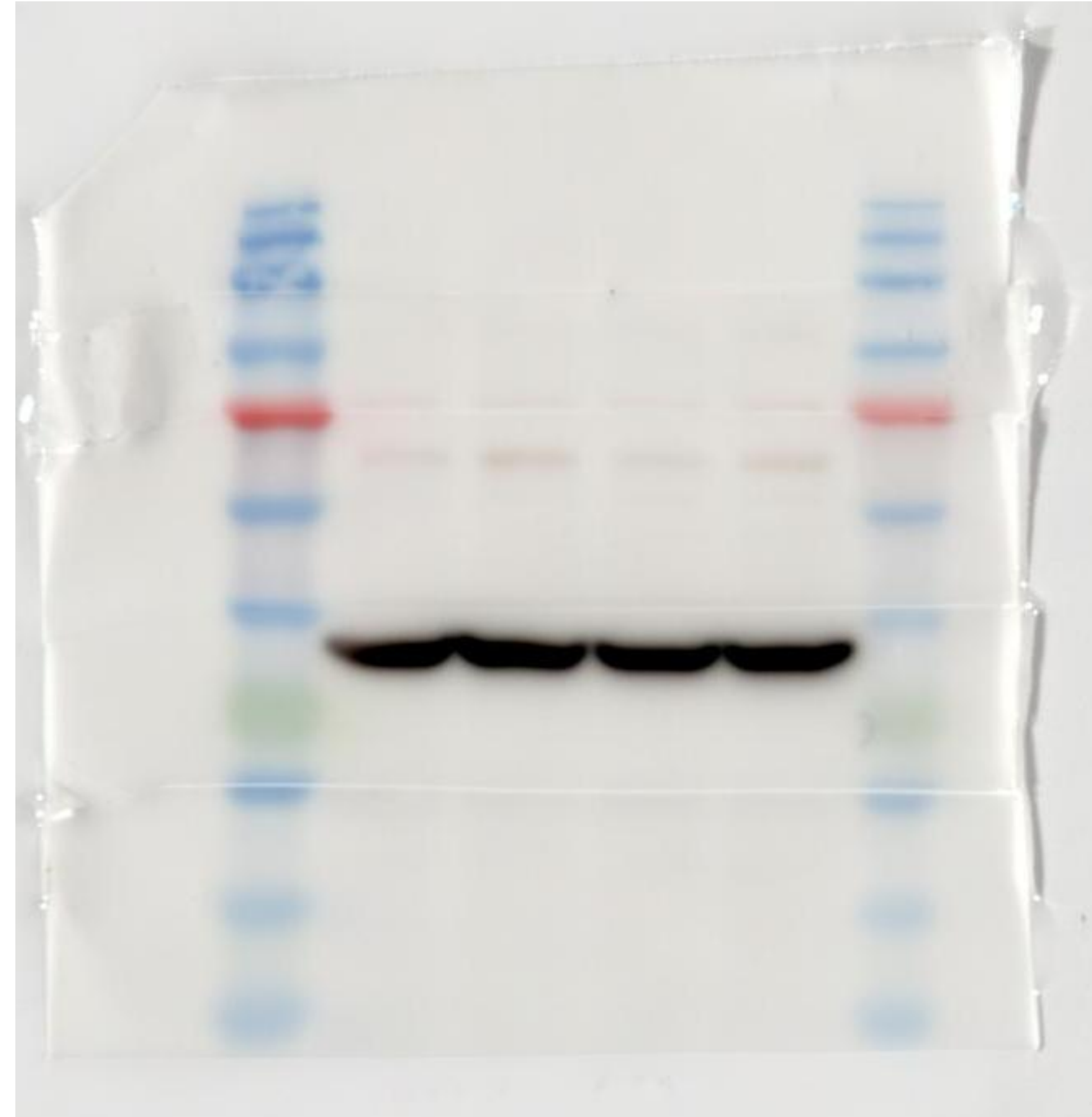

PI3K:85kDa original

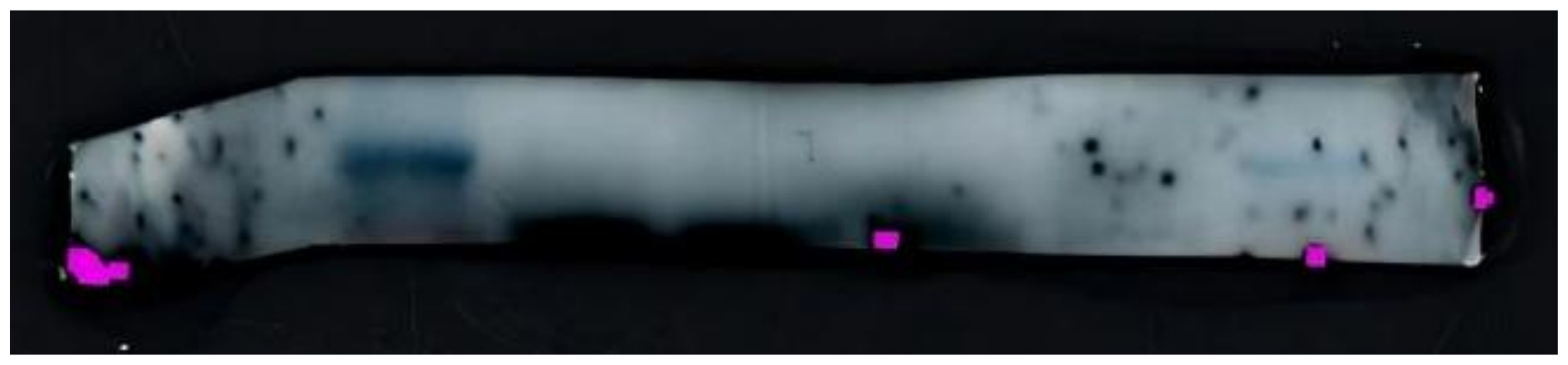

——75kDa

AKT:60kDa original

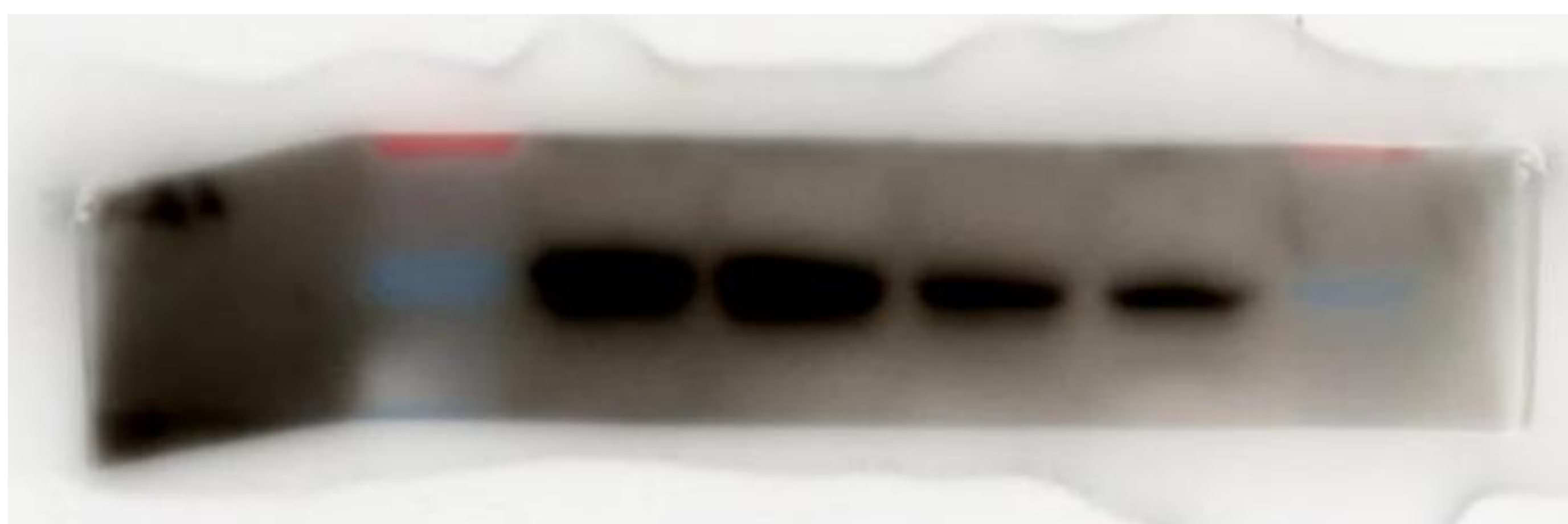

——60kDa

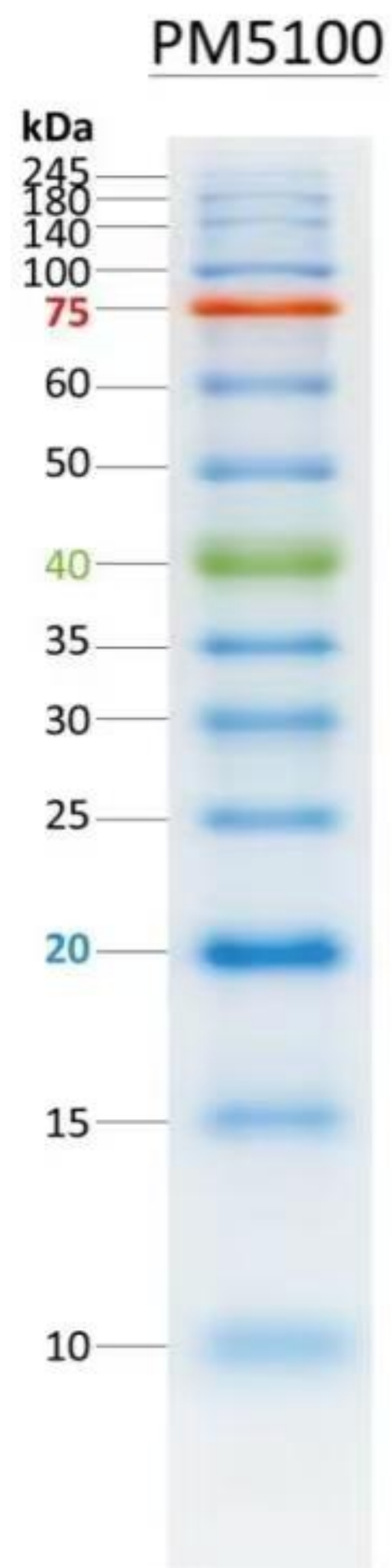

——35kDa

P-S6:32kDa original

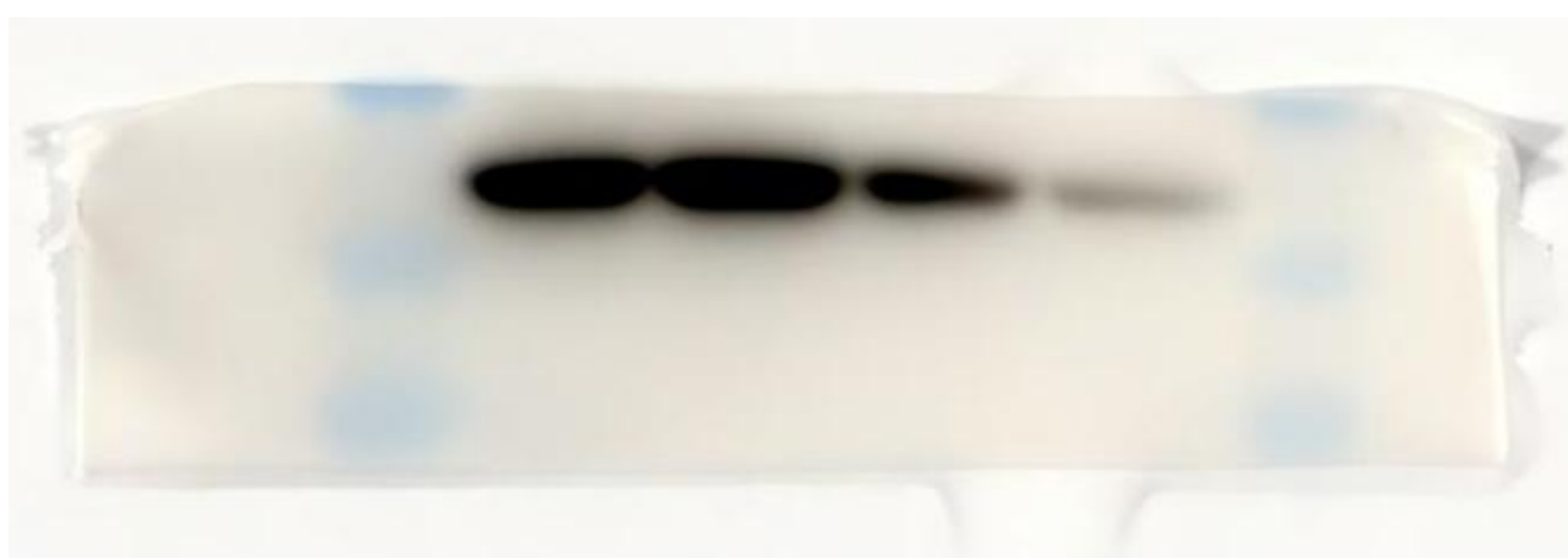

β-actin：42kDa original

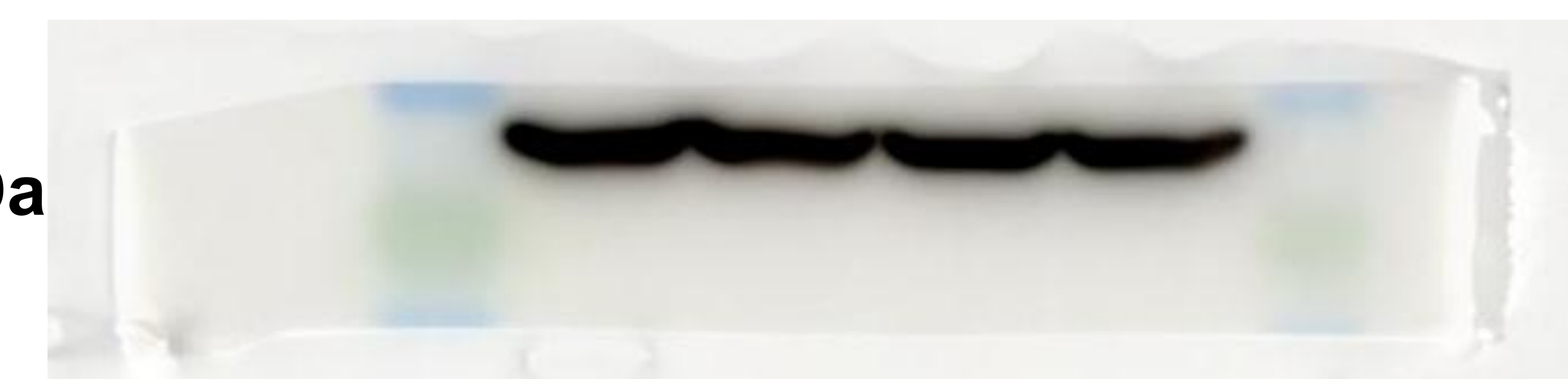

——40kDa

fig.7A-original

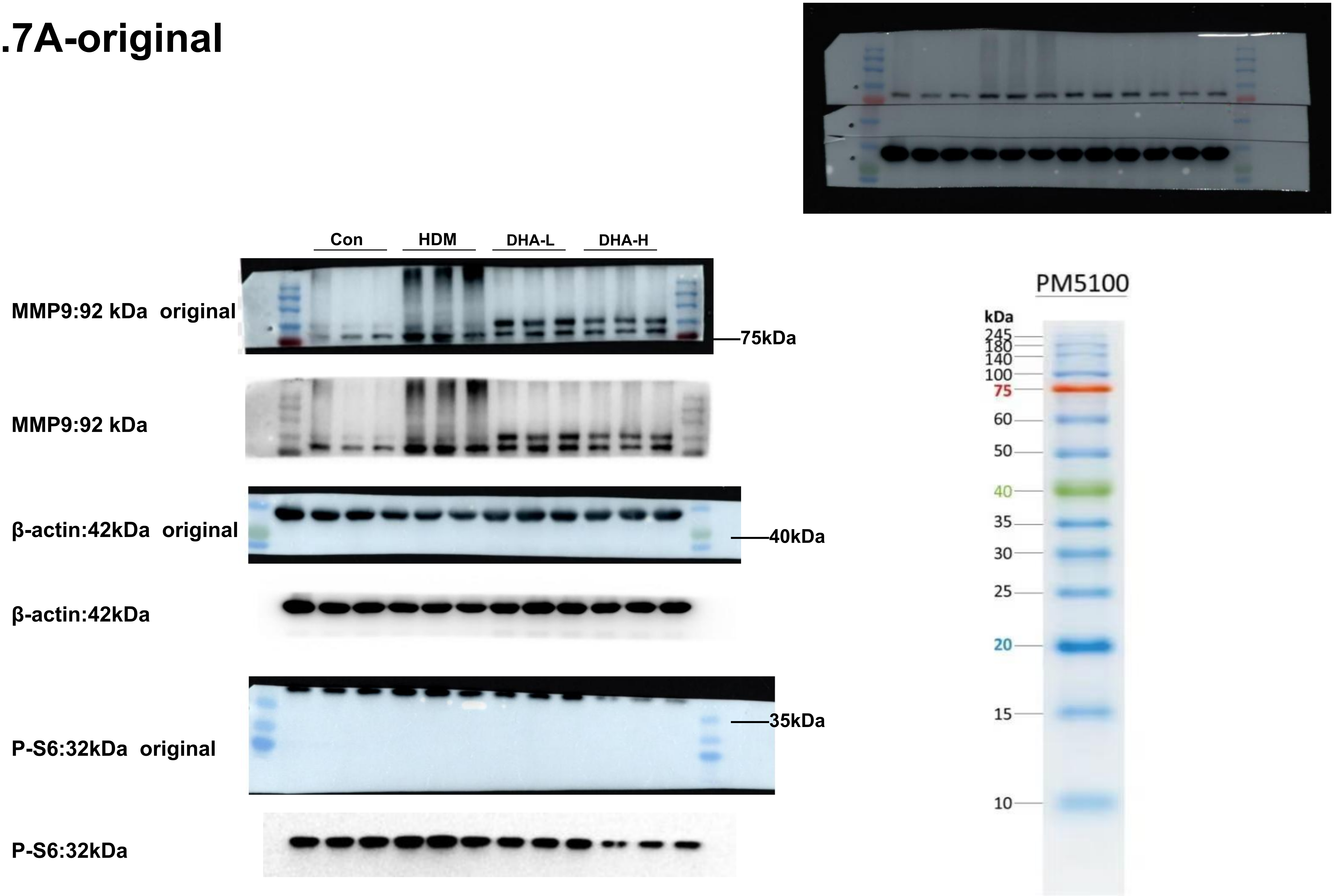

fig.7A-original

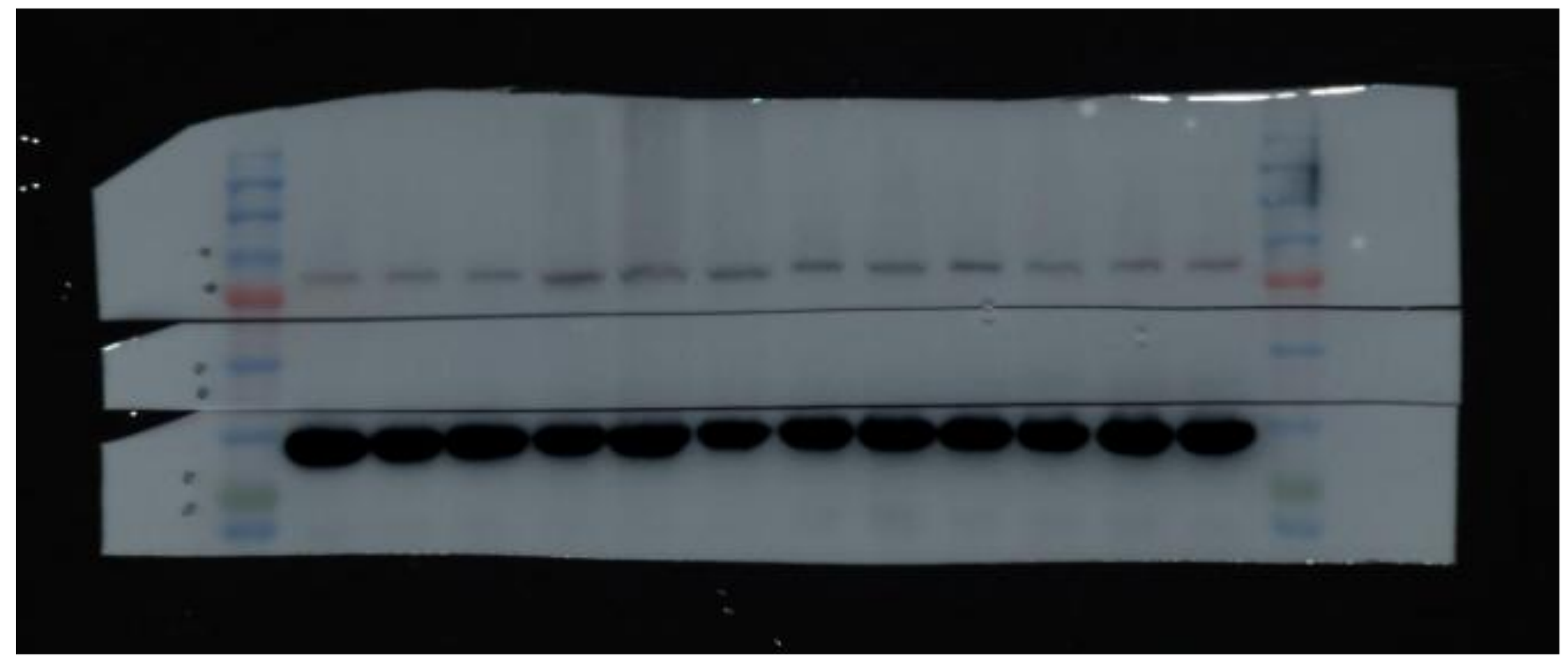

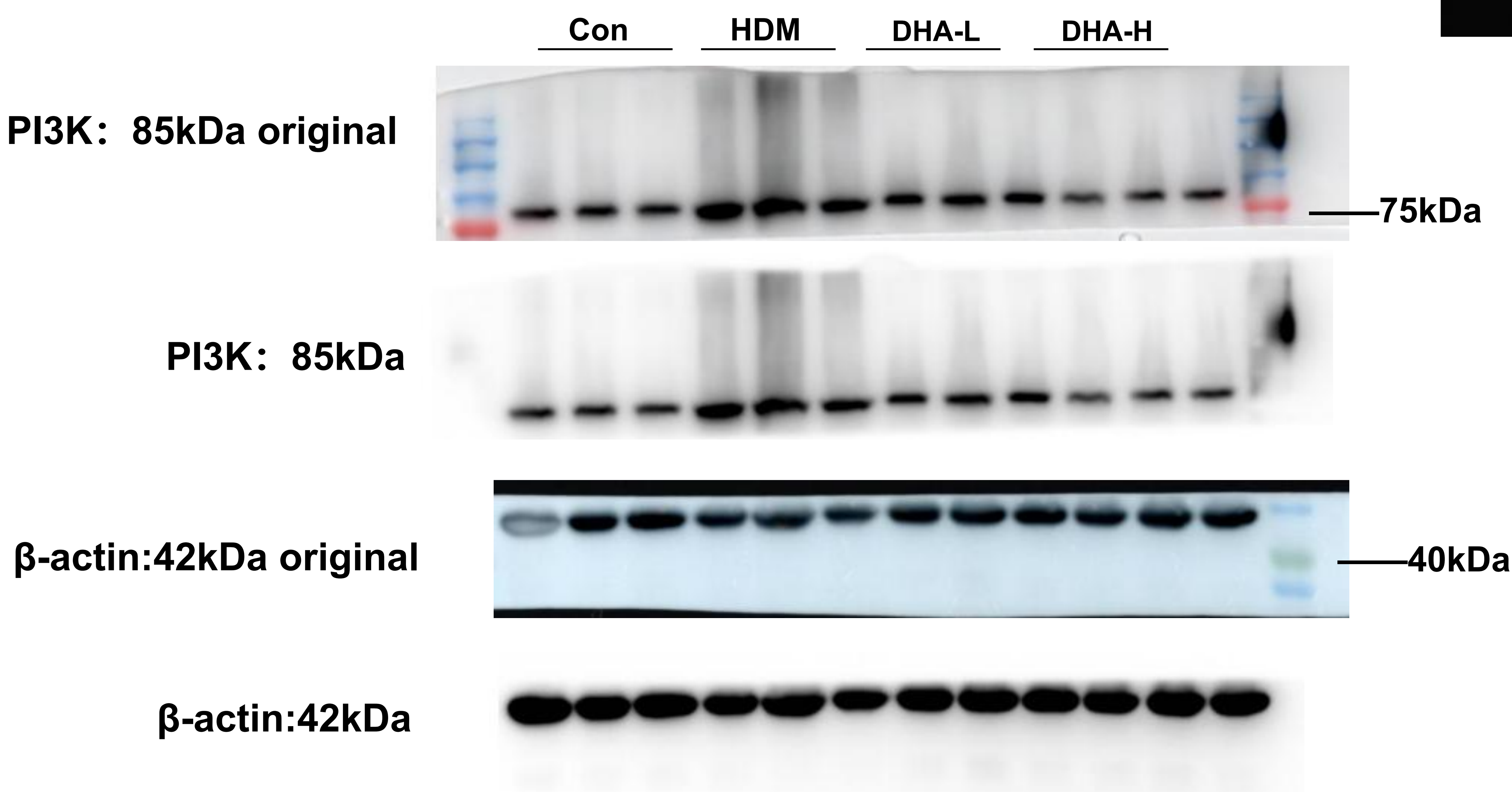

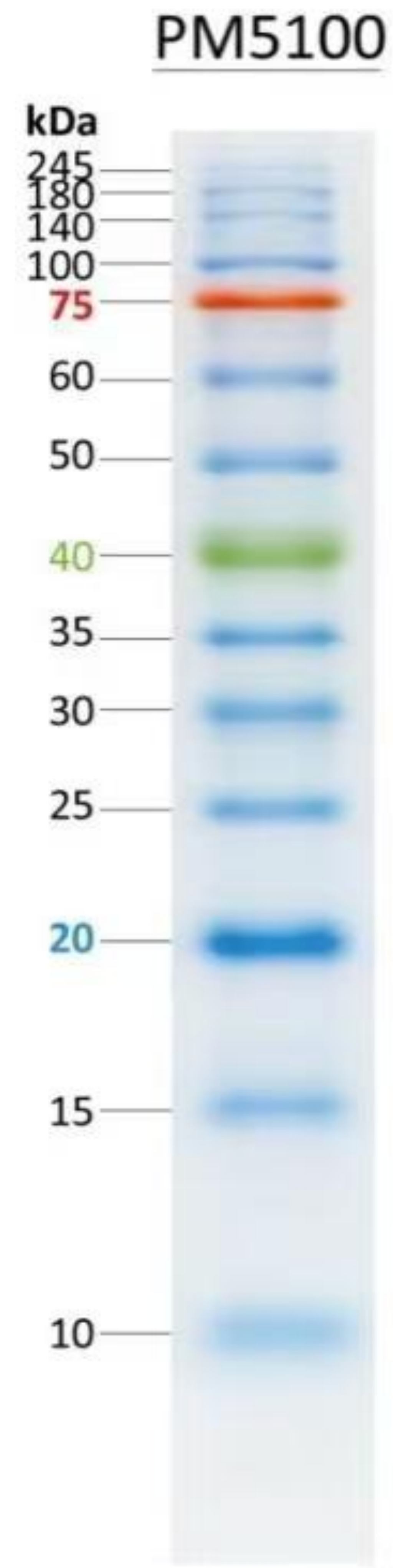

fig.7A-original

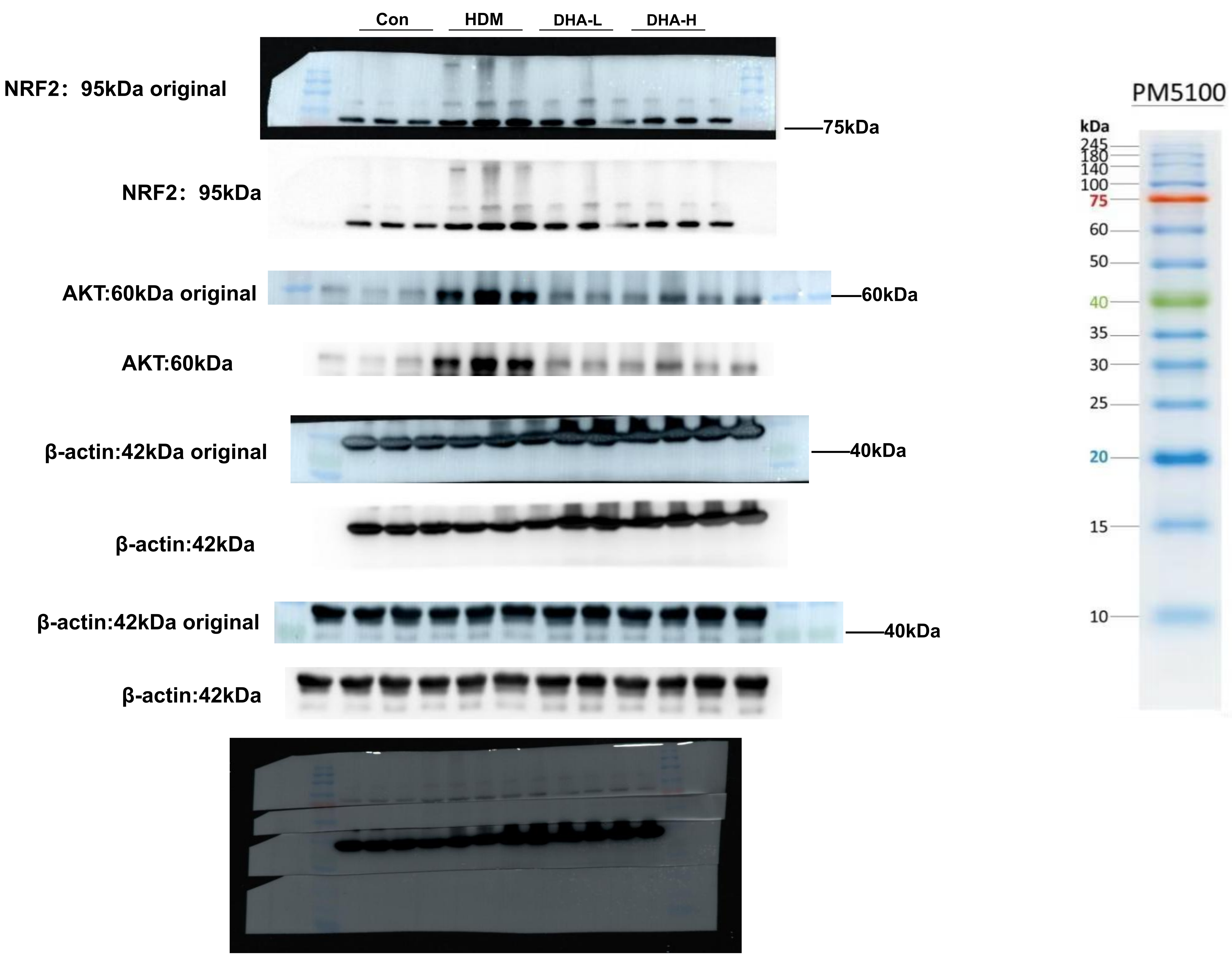

Supplement: Supplementary file 1 [file 12931_2026_3956_MOESM1_ESM.pdf]
